# Manufacturing, Processing, and Characterization of Self-Expanding Metallic Stents: A Comprehensive Review

**DOI:** 10.3390/bioengineering11100983

**Published:** 2024-09-29

**Authors:** Saeedeh Vanaei, Mahdi Hashemi, Atefeh Solouk, Mohsen Asghari Ilani, Omid Amili, Mohamed Samir Hefzy, Yuan Tang, Mohammad Elahinia

**Affiliations:** 1 Mechanical Industrial and Manufacturing Engineering Department, University of Toledo, Toledo, OH 43606, USA; omid.amili@utoledo.edu (O.A.); mohamed.hefzy@utoledo.edu (M.S.H.); mohammad.elahinia@utoledo.edu (M.E.); 2 Department of Materials and Metallurgical Engineering, Amirkabir University of Technology, Tehran 1591634311, Iran; mahdi.hashemi@aut.ac.ir; 3 Biomedical Engineering Department, Amirkabir University of Technology (Tehran Polytechnic), Tehran 1591634311, Iran; atefeh.solouk@aut.ac.ir; 4 School of Mechanical Engineering, College of Engineering, University of Tehran, Tehran 1439957131, Iran; mohsenasghari1990@ut.ac.ir; 5 Department of Bioengineering, University of Toledo, Toledo, OH 43606, USA; yuan.tang@utoledo.edu

**Keywords:** self-expanding metallic stents, shape memory alloy, additive manufacturing, biomimicry, biomaterials, patency rate

## Abstract

This paper aims to review the State of the Art in metal self-expanding stents made from nitinol (NiTi), showing shape memory and superelastic behaviors, to identify the challenges and the opportunities for improving patient outcomes. A significant contribution of this paper is its extensive coverage of multidisciplinary aspects, including design, simulation, materials development, manufacturing, bio/hemocompatibility, biomechanics, biomimicry, patency, and testing methodologies. Additionally, the paper offers in-depth insights into the latest practices and emerging trends, with a special emphasis on the transformative potential of additive manufacturing techniques in the development of metal stents. By consolidating existing knowledge and highlighting areas for future innovation, this review provides a valuable roadmap for advancing nitinol stents.

## 1. Introduction

In the last 30 years, many treatments, particularly surgical ones (open/laparoscopic), have been employed to primarily treat benign/malignant gastrointestinal obstructions [1,2]. Coronary artery disease is a leading cause of death worldwide, which is characterized by the narrowing of arteries due to the buildup of plaque (atherosclerosis). Various procedures are available to occlude arteries, such as bypass surgery, balloon angioplasty, and stent placement [3]. Self-expandable metallic stents (SEMSs) have been increasingly used (fluoroscopically or endoscopically) as a bridge to surgery for patients suffering from advanced or metastatic disease (e.g., gastric, pancreatic, colonic, colorectal cancer, and atherosclerosis) [1,4,5,6,7]. SEMS placement, as a non-surgical, palliative, minimally invasive, and safe procedure, is associated with significantly lower mortality and morbidity compared to palliative surgical treatments. It has already been linked to higher clinical success rates, shorter recovery times, and therefore reduced hospital stays [1,4,5,7,8,9]. The implantation of SEMSs can also eliminate/palliate symptoms, such as pain; prevent aspiration-associated infections; reduce overall costs; prevent recurrent laparotomies; and ultimately improve the quality of life [1].

Given the challenges in diagnosing malignant biliary obstruction in its early stages, particularly in older patients, surgical treatment options are often not viable due to factors such as the patient’s age, the tumor’s location, or the presence of other systemic diseases. Therefore, transhepatic or endoscopic stent insertion has been considered a primary palliative treatment to relieve biliary or colorectal obstructions so as to improve the quality of life [10,11,12,13]. Stenting is widely employed as a typical treatment for atherosclerosis to restore the patency of narrowed/clogged blood vessels [14,15]. Since the elemental stages of stent invention, stenting has been rapidly developed to overcome the limitations of balloon angioplasty and is widely used in interventional therapy [16,17]. Bare-metal stents and drug-eluting stents have been developed to reduce restenosis in coronary arteries [3].

**Figure 1:**
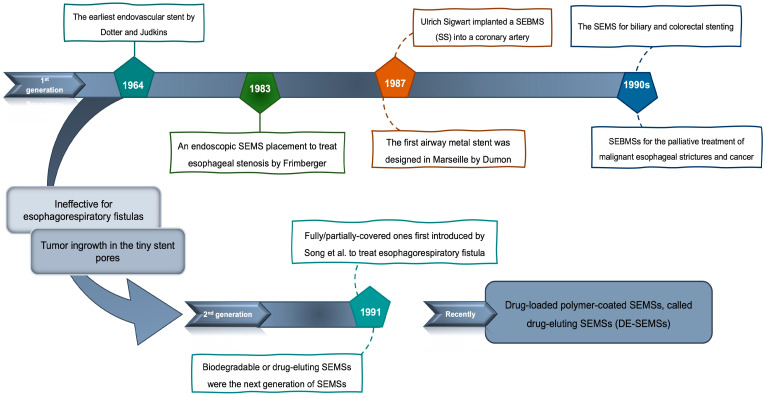
A timeline for the development of the stents from early 1960s. The evolution from the first to the second generation is depicted.

In 1964, Charles Dotter and Melvin Judkins produced the earliest endovascular stent [18]. In 1983, Frimberger became the first to place an endoscopic SEMS to treat esophageal stenosis. Currently, stents are widely used to treat various esophageal diseases [19]. Indeed, self-expanding bare-metal stents (SEBMSs) are the first generation of SEMSs [20]. In 1987, Ulrich Sigwart implanted a SEBMS into a coronary artery to solve its obstruction [21]. At the same time, the first airway metal stent was designed in Marseille [2]. SEMS for biliary and colorectal stenting were introduced first in the early 1990s [4,6,22]. Since then, SEBMSs have been increasingly used for the palliative treatment of patients with malignant esophageal strictures and cancer [20,23,24,25,26]. Despite the overall successful placement of these stents, they were ineffective for treating esophagorespiratory fistulas and allowed for tumor ingrowth in the tiny stent pores, leading to recurrent dysphagia (difficulty in swallowing). The second generation of SEMSs is the fully/partially covered ones first introduced by Song et al. in 1991 to treat esophagorespiratory fistula. In a nutshell, SEMSs, in terms of using coating, have already been placed into the body uncovered (bare) or fully/partially covered [5,20]. Subsequently, retrievable fully covered SEMSs were designed to allow removability and therefore employed for patients with benign esophageal strictures. Biodegradable or drug-eluting SEMSs were the next generation of SEMSs that emerged to solve the problems of stricture recurrence after covered SEMS removal and to prevent granulation tissue formation at the end of a SEMS [20]. Recently, drug-loaded polymer-coated SEMSs, called drug-eluting SEMSs (DE-SEMSs), have been employed [27]. This evolution of stent applications is summarized in Figure 1. The global market for SEMSs was valued at approximately USD 3.22 billion in 2022. It is projected to grow at a compound annual growth rate (CAGR) of around 4.0% from 2023 to 2030 [28]. Key factors driving this growth include the rising prevalence of cardiovascular diseases, technological advancements in stent design and materials, and an increasing aging population.

This paper provides a comprehensive overview of the current practices and research across various areas of self-expanding metallic stent, including design, simulation, biomimicry, and patency, along with the challenges and existing approaches. In particular, the potential benefits of additive manufacturing techniques for metal stents are explored.

## 2. Metallic Stents

### 2.1. Materials for Stents

Stents are fundamentally made from metals, polymers, or their composites (hybrid) [2,29]. Compared to polymer stents, metal stents have higher patency rates and larger tube diameters as advantages but higher costs as disadvantages [29]. Metallic stents are produced from different elemental shapes of metals, including sheet, ribbon, wire, and tube [30,31].

**Figure 2:**
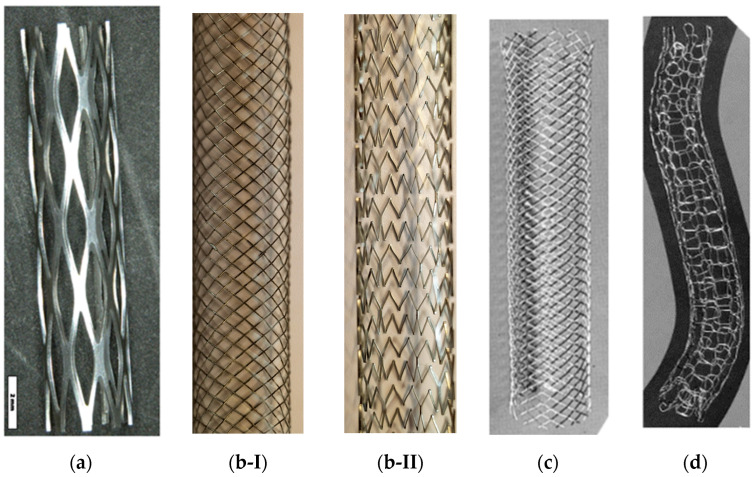
(**a**) Mg alloy stent. Reproduced from open access journals [40] (**b-I**) braided NiTi stent, (**b-II**) laser cut NiTi stent. Reprinted with permission [41]. Copyright 2021, Elsevier. (**c**) Cobalt alloy (**d**) Tantalum alloy stent. Reprinted with permission ([42]). Copyright 2009, Taylor & Francis.

Metallic stents exhibiting enhanced mechanical and corrosion properties are made of nitinol [4,22,32], stainless steel (SS) [4,22,33,34], Elgiloy [4], pure iron, Ti, Mg, Co-Cr [35,36,37], Pt-Cr, Pt, Ta, Zn, Pt-Ir alloys [21], and Fe-Mn alloys [30,38,39]. Nitinol (Ni_55_Ti_45_), due to its shape memory and superelastic behaviors, has been the most widely used material in stent manufacturing [30,31]. Indeed, recently, nitinol stents have largely replaced those made from other alloys, owing to nitinol’s improved elasticity, conformability, and compatibility with magnetic resonance imaging [4]. SS316L and Co-Cr alloy are commonly used due to their higher corrosion resistance through forming a stable Cr_2_O_3_ passive layer on the stent surface. However, Co-Cr alloy is nonmagnetic and, compared to SS316L, has a higher strength, density, corrosion resistance, and fatigue life, as well as better radiopacity. Indeed, the demand for producing thinner stents from materials with higher mechanical strength, such as Co-Cr, Pt-Cr, Pt-Ir, and Co-Ni alloys, is increasing. On the other hand, releasing Ni, Cr, and Mo ions from this material into the body causes allergic reactions [30,31]. Stents have already been manufactured from pure Fe, Mg, Ta, and Ti; however, just Fe and Mg stents have been extensively produced. Unlike the better biocompatibility, corrosion resistance, and visibility during deployment (under X-ray fluoroscopy) of Ta stents compared to SS316L stents, they could not be commercial due to their low strength and high recoiling percentage [30,31]. Meanwhile, Ti stents with excellent biocompatibility could not be commercially utilized due to their low ductility. However, considering the superior properties of Ta and Ti, they have already been employed as coatings on SS316L stents [30]. A few of these stents are shown in Figure 2.

Self-expanding stents, intrinsically showing shape memory and superelastic behaviors, can be produced with smaller diameters at room temperature, deploying themselves to the size of the vessel diameter at body temperature [31]. Self-expanding or self-expandable stents can be made from metals or plastic [20]. SEMSs and self-expanding plastic stents (SEPSs) have proven to be superior to rigid plastic stents because of their fewer complications, lower mortality, and better pain relief. However, SEPSs have previously shown higher migration risks than SEMSs [26]. Self-expanding metallic stents are made from 316 SS, Ni-Co alloy (MP35N), and nitinol [43]. Ni-free Zr-based bulk metallic glasses (BMGs), such as Zr_60_Fe_10_Cu_20_Al_10_, have recently drawn increasing attention as an alternative for producing SEMSs due to their superior hardness, strength, fracture toughness, and corrosion resistance; the absence of toxic elements (e.g., Ni and Be) in their composition; and their initial cytocompatibility with bone-marrow mesenchymal stem cells [14,15,44]. A comparison of these materials is presented in Table 1.

### 2.2. Classification of Stents

**Figure 3:**
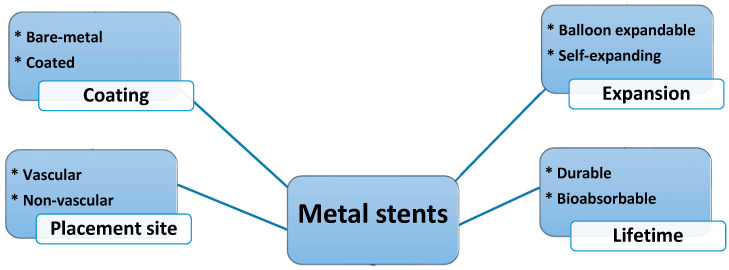
Classifications of metallic stents based on different terms.

As observed in Figure 3, metallic stents can be categorized based on different terms. Metal stents, in terms of expansion, are classified into two types: balloon-expandable stents (e.g., Co-Cr and SS) and self-expanding stents (e.g., nitinol) [3,17]. The advantages of a self-expanding stent compared to an expandable balloon include less damage to the vessel due to its lower pressure while dilating and its applicability for complex vascular lesions without using a balloon [46].

Metal stents can also be categorized as vascular or non-vascular. Vascular stents include coronary, carotid, and peripheral stents, while non-vascular stents include airway, ureteral, prostatic, esophageal, and biliary stents [2,47,48].

Metallic stents, in terms of using coatings, have already been inserted into the body coated (by fluorinated polymer, fiber, or natural coatings) or uncoated (i.e., bare metals) [49,50], e.g., coating the outside surface with heparin-bonded expanded polytetrafluoroethylene [17]. They are also called covered or uncovered stents [11,12]. The coating can be deposited through different methods, such as magnetron sputtering [21]. Previous studies have shown that uncovered SEMSs suffer from complications, like bleeding, fistulae, and stent ingrowth (i.e., tissue growth into the stent mesh), leading to new strictures or occlusions [27,51].

Although bare-metal stents (BMSs) are frequently used owing to their radiopacity, proper mechanical properties, fast/straightforward laser cutting, and low cost, their usage still suffers from in-stent restenosis (ISR) or lumen encroachment. However, thinner BMSs show lower restenosis rates. Recently, BMSs have been mainly made of Co-Cr and Pt-Cr alloys rather than stainless steel (SS) due to their flexibility in designing thinner struts [30]. Although BMSs reduce vessel blockage, they may suffer from high restenosis rates. To overcome this problem, drug-eluting stents (DESs), metallic stent coated with antiproliferative drugs, was introduced. DESs can also reduce neointima hyperplasia and target lesion revascularization (TLR), resulting in long-lasting vessel wall healing [52]. Drug-eluting stents gradually release the drug (e.g., sirolimus, paclitaxel, dexamethasone, actinomycin, and beta-Estradiol) into coronary arteries to limit cell proliferation and prevent fibrosis. Although DESs, compared to BMSs, have a lower risk of further revascularization, they are more expensive, cannot avoid myocardial infarction in randomized trials, and suffer from late stent thrombosis. Indeed, BMSs and DESs show high restenosis and thrombosis rates, respectively [30].

From another point of view, we can also categorize the stent materials into durable and non-bioabsorbable/fully bioabsorbable stents. Bioabsorbable stents, also known as bioresorbable or biodegradable, are a new generation of stents that can slowly dissolve in the body, made of biodegradable polymer or absorbable metals [31,53,54]. Employing biodegradable stents promotes several drawbacks of permanent metallic stents, including long-term endothelial dysfunction, slow re-endothelialization, thrombogenicity, and local chronic inflammation. The advantages are the possibility of using them in kids, no need for further surgery, no subsequent bleeding, and minimizing the risk of restenosis and late thrombosis [30].

Although biodegradable stents with superior mechanical and corrosion properties have already been made from Zn, Mg, Fe, and Fe-Zn, their production from stainless steel, Co-Cr alloys, or nitinol is still under investigation [14,21,30]. However, in Fe-based biodegradable stents, Fe^2+^ and Fe^3+^ ions decrease the proliferation of smooth-muscle cells and inhabitation of neointimal hyperplasia. Moreover, the degradation products of Mg- and Zn-based stents are toxic. Therefore, the composition of biodegradable stents should be modified to optimize and balance their biocompatibility and the mechanical/corrosion behaviors of the alloys [30].

**Figure 4:**
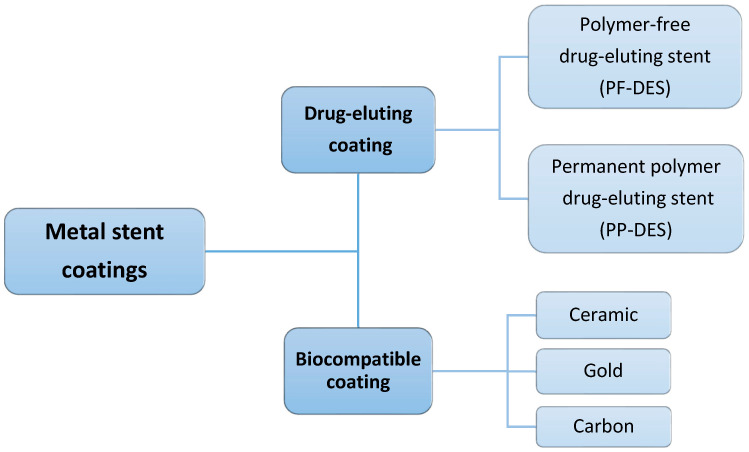
Classifications of coatings used for metal stents.

As seen in Figure 4, metal stents can be coated by drug-eluting or biocompatible materials [49,50].

## 3. Manufacturing Processes

Conventional stent manufacturing methods include casting, electroforming, etching, micro-electro-discharge machining, and laser cutting [19,30,52]. These methods are described in the following subsections.

### 3.1. Conventional Manufacturing

#### 3.1.1. Primary Manufacturing Processes (Mother Tube/Sheet Fabrication)

##### Casting

The welded–redrawn, or seamless, metallic tube is produced from cast ingot or sheet using forming and welding processes. Later, the tube is cut using the laser-cut process and then annealed [30,39]. Equal channel angular processing (ECAP) is a method that uses severe plastic deformation (SPD) to induce structure refinement, therefore producing ultrafine/nano-structure stents with improved strength and corrosion behaviors [30,55,56]. Recently, researchers have introduced novel ECAP with modified/different dies showing enhanced process performance, including tubular channel angular pressing (TCAP), parallel tubular channel angular pressing (PTCAP), and cyclic extrusion compression angular pressing (CECAP) [57]. The casting process is followed by the forming processes mentioned below to manufacture the micro-tube of stents [58]:

Hot extrusion: Hot extrusion is employed to directly/indirectly extrude large as-cast ingots and convert them into small, seamless, and thin-wall tubes at elevated temperatures [58,59].

**Figure 5:**
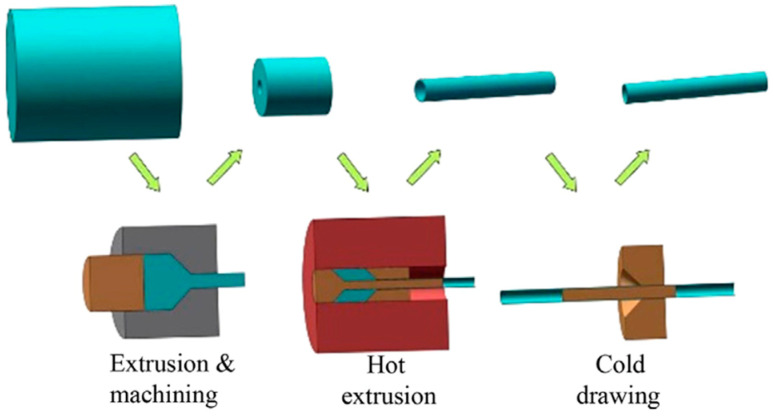
Schematic illustration of hot extrusion and cold tube drawing processes.

Cold tube drawing: Cold tube drawing generates near-net-shaped stents with enhanced mechanical performance from hot-extruded tubes [58,60]. Schematics of hot extrusion and cold tube drawing processes are illustrated in Figure 5.

After forming the sheets into tubes, tubular stents will generally be laser-cut and annealed [30].

##### Powder Metallurgy (PM)

PM is a near-net-shaped, reasonable-cost, and high-productivity manufacturing technology capable of producing small, complex, and high-performance samples with low impurities, uniform microstructure, and high densities. Afterward, the precursors prepared by PM can be subjected to hot extrusion or cold rolling to produce mother sheets/tubes. The properties of the final product are influenced by the size, morphology, and composition of metal powders. The processing parameters such as the pressing load and sintering atmosphere also affect the fabricated part. Mechanical alloying of powder particles using a ball mill creates samples with smaller particle sizes, lower porosity, and therefore a lower corrosion rate. Nitinol stents manufactured using spark plasma sintering of ball-milled powders have already shown a maximum radial force in expansion three times higher than commercially cast ones [30].

Conventional sintering (CS): Many researchers have previously produced Fe-Mn cardiovascular stents using CS for 15 min in a vacuum furnace [30,38,39].

Spark plasma sintering (SPS): Ni-Ti vascular stents have been formerly fabricated using the SPS process, followed by hot extrusion [30].

Metal injection molding (MIM): The MIM method can produce thin-wall tubular stents with porous structures suitable for drug reservoirs [30].

##### Electroforming

**Figure 6:**
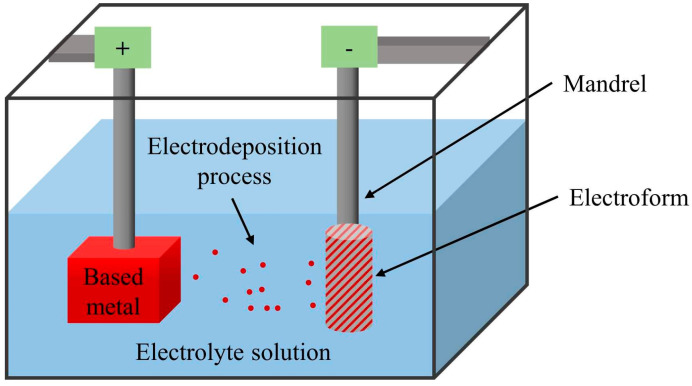
A schematic illustration of the electroforming method for manufacturing pure iron stent. Reproduced with permission [30]. Copyright 2022, Elsevier.

Electroforming is a thin atom-by-atom deposition of a metal on the surface of a conductive mandrel. Indeed, the metallic ions released from the anode into the solution are deposited onto the mandrel surface as the cathode. Once the desired thickness is deposited, the mandrel is detached, and the stent is produced using a laser cut. A simple schematic of the electroforming process used for manufacturing iron stents is illustrated in Figure 6. The involved parameters in this process, such as current density, electrolyte composition, pH, and temperature, affect the microstructure of the fabricated stent and can be tuned to obtain desired properties. Electroforming is a perfect and low-cost method for precise additive manufacturing of thin-wall and tubular products with complex geometry. Compared to casting and powder metallurgy methods, the electroforming process is capable of producing larger ingots [30,39,61,62].

#### 3.1.2. Secondary (Complementary) Manufacturing Processes

These processes are not individually complete and primary manufacturing processes but are considered part of the stent fabrication process.

##### Cutting Methods

Photochemical etching:

Photochemical etching, or photochemical machining (PCM), is a simple, rapid, low-cost, and flexible method for stent manufacturing, with no residual stress and burrs. In this method, the sample is coated with a photomask, and then the material is removed layer-by-layer via chemical reactions, using the proper etchant [30]. This method creates stent patterns on a sheet, followed by laser welding [21,40,63]. The stents can be produced with the precision of a few microns. However, the PCM is not suitable for manufacturing 3D complex products, and the coating on the stent surface may be non-uniform.

Micro-electro-discharge machining:

Micro-electro-discharge machining (μEDM) is a cutting method capable of fabricating stents with high surface quality and dimensional accuracy. In this method, the sample and a microscopic electrode are held in dielectric fluid, and the sample is cut by employing electrical pulses between the electrode and sample [30]. In the fabrication of stents, the patterns are created either on a cylindrical tube or a sheet (followed by folding and welding the patterned sheet). By controlling the discharge energy of the process, stents with high flexibility and thin walls can be produced [21,30,64,65].

Water-jet machining:

This method is a fast and economical cutting method in which patterns are cut from thin sheets using a focused jet of water with no heat-affected zone (HAZ) to create small stent-like structures. As a disadvantage, the produced stents have rough surfaces with low-quality cutting edges. HAZ is the area in the part that is exposed to a high amount of energy and a high temperature during the manufacturing process in welding and AM techniques. This high energy can change the microstructure and properties of this area and cause composition change that is crucial in the case of NiTi. The thermal cycle in HAZ can cause residual stress that can lead to warping and crack formation. These changes in properties and microstructure make HAZ an undesired phenomenon in fabrication [30].

Micro-milling:

Micro-milling is a microscale machining method that has already been employed to cut Mg-alloy tubes into stents. Although this method has high cutting efficiency and accuracy, it leaves some burrs at the stent edge, which may require some post-treatment [66,67].

Laser cutting:

Due to the high fabrication speed/precision/quality and low cost of laser cutting, it is the most used process to cut the thin-wall tube into a mesh-like stent [30,68].

Stents can be cut by different types of lasers, including the Nd: YAG laser, the fiber laser, and laser microjets [30,69]. The narrower kerf width of the fiber laser (12 μm) rather than the Nd: YAG laser (20–30 μm), attributed to its lower heat input, results in smaller HAZ, lower thermal distortion, and a more accurate stent profile. The Nd: YAG laser, with ultra-short nanosecond pulses and a high pulse repetition rate (e.g., Q-switched laser), is the most common laser used for stent cutting. Indeed, the short pulses of the laser result in a narrower kerf width, lower heat input, reduced HAZ, and economic viability. Laser microjets (e.g., a water jet-guided Nd: YAG laser or femtosecond laser) have already been employed to produce stents with no/little debris and thermal damage. Although the femtosecond laser is costly, it is capable of cutting materials with nanometric precision and lower damage to the surrounding area, leading to faster patient recovery [30].

Laser cutting can be employed in two stages:

Cutting the cast sheet;

Cutting the tube to produce a slotted tube.

##### Laser Welding

Laser welding joins the wire in the stents. It can also weld stent patterns already produced by the photochemical etching process [40,63]. In braiding, the intersection points of wires can be welded together via laser welding [30]. Wire-to-wire joining by welding process has recently become a good alternative for laser cutting to avoid the formation of a wide HAZ degrading the microstructure and mechanical properties of the stent. In addition, more than 90% of the initial material is removed during the stent manufacturing through the laser-cutting process, leading to an increase in manufacturing costs.

##### Coating

Coating complementary processes can be considered as part of the manufacturing methods of stents. Indeed, depositing coating onto the stent surface (e.g., tantalum coating on SS316L) protects them from corrosion or any interaction in contact with the surrounding biological environment, leading to the enhanced mechanical properties and lifetime of stents [31,70].

Stent coatings can be categorized as passive and bioactive. Passive coatings on bare metal protect them from any interaction between the stent and the biological environment. Passive coating materials such as polytetrafluoroethylene (PTFE), polyethyleneglycol (PEG), chitosan, and dextran show antifouling properties to avoid the adhesion of proteins/cells on the stent surface. Moreover, inorganic coatings such as diamond-like carbon, iridium oxide, titanium–nitride–oxide, and silicon carbide can limit the metal ion release. This type of coating can also be employed to facilitate the handling of stents during implantation (e.g., gold coatings on SEMSs to provide radiopacity). Bioactive coatings protect the stents by interacting with them to avoid disruptive phenomena like thrombus formation. Bioactive coating materials show anti-thrombogenic properties and explore proteins, polysaccharides, and phospholipids to the proliferation of endothelial cells [31].

The coating can be deposited on metal stents (e.g., nitinol) using different methods, such as the electrospinning (ES) process, plasma treatment, or laser evaporation [31,71].

### 3.2. Novel Manufacturing

Novel manufacturing techniques for fabricating stents are weaving (which is categorized into knitting and braiding) and additive manufacturing.

#### 3.2.1. Weaving

Weaving or mesh-tube fabrication includes knitting and braiding methods, followed by welding for manufacturing mesh-tube stents [30].

##### Knitting

In this method, a woven (also termed “hooked wire” or “D-weave”) structure is created by hooking wires around each other using a mandrel [30,72]. As advantages of knitted structures compared to braided ones, knitted structures are more flexible and can be readily unraveled by pulling out one wire. In addition, the knitted stents show higher radial force (lower radial narrowing) but lower axial force due to wire junctions. The knitted stents have a lower bending stiffness and shortening ratio, and their compression resistance is insufficient. One of the shortcomings of this method is that the biomechanical limitations of a plain knit (PK) structure may cause clinical complications. Moreover, owing to the application of a PK in the artery, the mismatch between their longitudinal flexibility and radial compliance with the artery is challenging [30]. Knitted stents are further discussed in Section 4.

**Figure 7:**
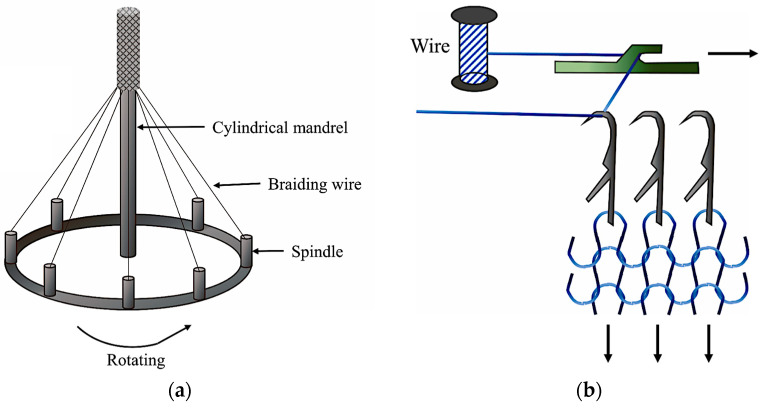
Schematic illustrations of textile construction methods for manufacturing metal stents: (**a**) braiding and (**b**) knitting. Reproduced with permission [30]. copyright 2019, Elsevier.

The application of bioresorbable helical stents is restricted due to their poor compressibility in an expansion system. Braiding and knitting are two textile manufacturing processes that address this problem due to their desired flexibility [30]. Schematic illustrations of braiding and knitting processes are shown in Figure 7.

Braiding:

In this method, a 3D woven (also termed “crossing wire” or “S-weave”) structure is created through a single strand of metal wire wrapped around a metal mandrel [30,73,74]. The stents produced by braiding (rather than other methods) show superior properties, such as high mechanical strength, torsional strength, flexibility, wear resistance, shape recovery, and good dimensional stability and compliance [30,68]. Moreover, braided stents are free of HAZs and can stretch up to twice their initial size, which is helpful for removing a stent. These stents should undergo shape setting to preserve their particular structure and electropolishing to obtain good surface quality. However, high axial rigidity and length variations in the braided stents; their shortening problems in neighboring tough tissues; and the sliding of wires, leading to inadequate radial support and restricted structural stability, can be considered as the main challenges of the stents. The braiding angle and number of wires per layer are key factors affecting the mechanical properties of braided stents. Braided stents are available in various designs and sizes for employment in different parts of the human body [30]. Some studies have been performed to enhance mechanical properties by design modification that are further discussed in Section 4.

#### 3.2.2. Additive Manufacturing

Additive manufacturing (AM), commonly known as 3D printing, is an advanced manufacturing method in which the sample is fabricated according to a designed model [21]. 3D printing, compared to conventional manufacturing, can produce parts with reduced cost and time but lower strength due to its layer-by-layer manufacturing process [30].

In addition to reviewing the work performed in additive manufacturing on self-expanding stents, we also review its benefits. 3D printing enables us to design stents with different cross-sections compatible with hemodynamics and vessel-wall behavior, resulting in reduced neointimal hyperplasia (NH), inflammation, and vascular injury [75].

One of the 3D-printing processes is selective laser melting (SLM), also called the laser powder bed fusion (LPBF) technique, in which the laser beam melts and fuses the powder particles [21,30]. In the SLM process, the tubular stents can be printed and then cut in one step using a laser beam [30]. The LPBF process helps designers produce long-duration stents with biomimetic design, improving blood-flow profiles and providing low shear stress [21,37,76,77]. Metal stents have already been made from SS316L [35,78], Co-Cr [35,36,37], and Ti alloys [79] using the LPBF process.

Various process parameters, including powder characteristics, laser power, scanning speed, hatch distance, layer thickness, and scanning strategy, can significantly influence the properties and performance of LPBF-fabricated NiTi. To achieve the desired performance in NiTi implants, precise control over energy density is crucial. For example, reducing energy density leads to increased porosity, which in turn decreases the overall density. Therefore, the energy density is an important factor to obtaining the desired properties. A major challenge in LPBF of NiTi is the reduction of Ni content, which can significantly impact the transformation temperatures. This depletion of Ni happens when higher energy densities are used [80]. 

Besides LPBF, directed energy deposition (DED) is another additive manufacturing process where energy source in the form of laser, plasma arc, or electron beam melts and fuses materials (in the form of powder or wire) as they are deposited. The energy source power, deposition rate, and feedstock flow rate are some examples of the process parameters that can be tuned to obtain the desired properties [81].

**Figure 8:**
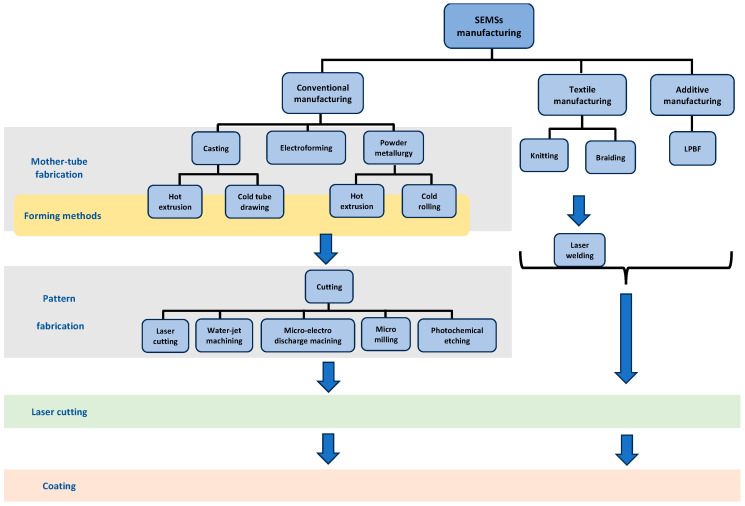
Manufacturing methods of SEMSs.

To create a better understanding, the manufacturing methods of SEMSs are illustrated and compared in Figure 8 and Table 2, respectively. Moreover, specifications of some industrial SEMSs produced using different manufacturing methods are listed in Table 3.

### 3.3. Post-Processing Techniques

#### 3.3.1. Laser Snipping (Cutting)

Laser snipping removes excess materials from stents fabricated from tubes to achieve the desired design. This high-precision method offers benefits such as minimal thermal distortion and smooth edges [35]. The laser parameters, including its power and speed, are controlled to ensure precision.

#### 3.3.2. Surface Modification

Surface modification of stents can be carried out through physical or chemical methods to improve stent biocompatibility and intimal healing, provide better control of the drug-release rate of the stent, and enhance the adhesion/proliferation of endothelial cells [21]. Optimizing the stent surface roughness can minimize the negative effect of stenting [35]. The hemocompatible surface of a stent enhances endothelialization, is biomimetic to replicate normal blood flow patterns, and inhibits intimal hyperplasia to achieve an antithrombosis and anti-restenosis outcome. Creating a micro-groove/pillar/lens array on the stent surface improves blood fluidity and compatibility after implantation, while inhibiting macromolecule adhesion in the blood [21].

##### Polishing

A surface chemical/electro/electrochemical/mechanical polishing process will be required prior to stent application [34,35,36,37]. Electropolishing can deposit a corrosion-resistant coating onto the surface of the stent, which enhances the surface quality and smoothness, leading to superior corrosion resistance and fatigue life. Moreover, electropolishing reduces contamination from foreign resources by decreasing free surface energy, resulting in minimizing thrombosis formation. Polishing under a magnetic field can improve the properties further. Electropolishing of nitinol stent reduces the nickel concentration on its surface [21,82].

#### 3.3.3. Heat Treatment

Heat treatment is performed on the stents for shape setting and improving mechanical properties. Annealing and aging are used to relieve internal stresses, enhance fatigue life and mechanical properties, stabilize transformation temperatures, and improve superelasticity. Mechanical properties are improved through precipitation hardening by formation of Ni-rich precipitates such as Ni_4_Ti_3_ and Ni_3_Ti in temperature range of 300–600 C. the size and distribution of precipitates depend on the temperature and time of heat treatment and the composition of the matrix [83].

## 4. Biomimetic Design

The selection of the form of a stent or stent design is a crucial factor influencing its performance. The diameter of the stents, the form of the struts, the width, length, and thickness of the struts, are all used to classify the stents. These designs are summarized in the following subsections.

### 4.1. Stent Basic Design

The first versions of stents were initially categorized into two main types of designs: slotted tube configurations, like the Palmaz stents; and coil configurations, like the Gianturco–Roubin Flex stent [84]. Slotted-tube designs offered strong radial support but were not very flexible, whereas coil designs were flexible but lacked radial strength. These conflicting design requirements led to the development of a diverse range of stent shapes in a competitive market, each aiming to strike the perfect balance between strength and flexibility [85]. As a result, stents can now be categorized into three primary groups based on their design: coil, slotted tube, and tubular mesh. Within these design categories, many stent designs can be developed by modifying factors like strut width, length, thickness, shape, or the overall stent diameter [84]. Numerous stent geometries have been explored and brought into commercial use to enhance the durability and performance of stents.

#### 4.1.1. Coiled Stents

Coiled stents are crafted from metallic wires or strips formed into a circular coil shape, representing the most basic form of stent design. Most coiled stents in today’s market are balloon-expandable and are frequently used in nonvascular applications such as prostate and urethral stenting. This preference arises from their exceptional flexibility and the ability to retrieve them after implantation. However, coiled stents have not been notably successful in vascular applications. This is primarily due to their large size when in a constrained state, limited radial strength, low expansion capability, and significant elastic recoil, as well as a heightened risk of restenosis [5].

#### 4.1.2. Slotted Tube Stents

These stents are constructed using metal tubes, and a stent design is created by laser cutting the material. The components of these stents are primarily aligned along the longitudinal axis and exhibit inherent rigidity along this axis. They represent a significant portion of the stents available in the market, characterized by their impressive radial strength but limited flexibility and deliverability [5].

#### 4.1.3. Tubular Mesh or Woven Stents

Woven stents are constructed by winding wires together to create a tube-like mesh structure. Woven geometries encompass a variety of designs made from one or more wire strands. This category includes both braided and knitted stents. Braided designs, known for their extensive coverage and minimal expansion during deployment, are more commonly used in self-expanding stents. However, these woven designs are employed in both self-expanding and balloon-expandable stent structures. These stents offer robust mechanical support to the arteries by covering the surface area with interwoven wires [30]. They are primarily intended for urological, gastrointestinal, and airway applications.

##### Fiber-Based (Fibrous) Stent

Stainless steel and other metallic materials are frequently employed to fabricate stents because of their favorable mechanical characteristics. Nevertheless, they come with drawbacks, including poor visibility in X-rays, susceptibility to corrosion, the potential for restenosis, and the risk of bleeding complications. To address these issues, researchers are currently exploring alternative materials, such as fibers, which offer unique advantages. These materials can be easily modified to enhance their compatibility with human tissues. Additionally, monofilament fibers possess exceptional mechanical properties, effectively enduring compression, tension, and bending forces. This makes them well-suited to meet the mechanical requirements of stents. The production of fibrous stents is achievable through techniques like knitting and braiding [86].

##### Braided Stent

Several key factors influence the mechanical properties of braided stents, including (a) their geometric configuration, (b) the properties of the individual monofilaments, (c) the braiding technique employed, and (d) the conditions during deployment.

An ideal braided stent should strike a balance between providing robust radial support and maintaining flexibility. However, it is often the case that enhancing radial support can compromise flexibility, creating a trade-off. Only some studies have explored methods to improve the radial support of braided stents without sacrificing flexibility. One approach uses composite materials, such as polymer/metal combinations, during braiding. Existing polymeric braided stents have faced a challenge where they tend to sacrifice too much flexibility to pursue improved radial support. This compromise can be detrimental when dealing with curved and circuitous peripheral lesions.

Additionally, it results in decreased stent flexibility and a higher axial elongation rate. While the diameter of monofilaments and the pitch angle during braiding influence stent mechanical properties to some extent, relying solely on these factors to enhance stent performance is not feasible. Furthermore, compared to conventional stents with larger monofilaments, mixed-braided stents that incorporate monofilaments of different diameters show a relatively small difference in radial support. This suggests that mixed-braided stents offer a potential avenue to enhance the overall mechanical properties of braided stents, holding promise for further research in this field [87].

##### Knitted Stent

Knitted textiles can be categorized into two fundamental types: warp knit, akin to tricot; and weft knit, resembling a hand-knit sweater. In warp knitting, the loops formed by each warp thread extend lengthwise along the fabric. In contrast, weft knitting involves the creation of loops by each weft thread, primarily across the fabric’s width [88]. Knitted arrangements possess a natural flexibility owing to their interconnected looped design. This inherent flexibility enables them to surpass the drawbacks associated with braided stents, such as limited flexibility and a tendency for the edges to fray.

Furthermore, in the event of a complication, a knitted stent can be easily removed in the form of a wire by straightforward unraveling [89]. Lin et al. explored using a blend of one-to-three biodegradable polyvinyl alcohol (PVA) yarns with different twist factors. This mixture gave rise to single-, double-, and triple-PVA twisted yarns. These twisted yarns were subsequently utilized to craft PVA vascular stents through braiding, warp-knitting, and weft-knitting techniques. The results indicated that the weft-knitted stent had a superior bending performance and a porous structure, facilitating nutrient exchange and waste discharge. It also provided space for cell migration and proliferation [90].

#### 4.1.4. Covered Stents

Self-expanding metallic stents have been developed to treat malignant or benign esophageal constrictions. However, a significant challenge in ensuring successful treatment is the excessive tissue growth that occurs in response to mechanical injury. To address this issue, fully or partially covered SEMSs have been devised specifically for esophageal strictures. These surrounding membranes serve to prevent excessive tissue growth around the wire meshes. Nevertheless, there remains a risk of granulation tissue forming at the exposed ends of the stent and tissue ingrowth through the disrupted covering [91].

### 4.2. Design of Patient-Specific Stents

Currently, the development of patient-specific vascular stents for individuals with vascular conditions is gaining significant attention in research. These stents are designed based on the unique shape of the patient’s blood vessels. The key feature of a patient-specific stent is its ability to conform to and replicate the exact shape of the patient’s blood vessel after it is deployed and undergoes deformation. This precise geometric alignment prevents or greatly diminishes the stress interactions between the stent and the vascular wall, ultimately leading to a significant reduction in in-stent restenosis [92].

#### 4.2.1. Stent Strut Design

The connection or bridge is a crucial element influencing the longitudinal flexibility of the stent. Concerning the design of “bridge” vascular stents, researchers have emphasized that the geometric characteristics of these connections dictate the stent’s mechanical behavior. Currently, vascular stents employ various bridge shapes, including L-shaped, N-shaped, V-shaped, and S-shaped bridges. In an assessment of the mechanical properties of stents featuring L-shaped, V-shaped, and S-shaped bridges, it was observed that stents with L-shaped bridges exhibited the lowest axial stiffness [92].

#### 4.2.2. Cell Design

##### Closed-Cell

In a closed-cell design, all the internal bending points of the structural components are linked together using bridging elements. As a result, closed-cell stents are less flexible but offer greater radial strength compared to similar open-cell designs. Closed-cell stents are also more resistant to the growth of tumors or excessive tissue growth inward, in contrast to open-cell stents. Consequently, this stent type tends to exhibit a longer duration of patency [93].

##### Open-Cell

In open-cell-designed stents, the bridging elements do not connect all or any internal bending points within the structural components. Instead, there are periodic connections from peak to peak, from peak to valley, and from mid-step to mid-step. These unconnected structural components contribute to the stent’s longitudinal flexibility, making them more pliable but with lower radial strength and a higher tendency for plaque prolapse [93]. Stents with wide or open cells have been specifically engineered to achieve several benefits, including reducing the proportion of metal surface area, improving access to side branches, enhancing conformability, minimizing arterial injury, and reducing neointimal reaction. When it comes to the shortening ratio of the stent before and after deployment, the open-cell design, often featuring a laser-cut structure, outperforms the closed type, as its shortening ratio is zero [94].

##### Helical Patterns

**Figure 9:**
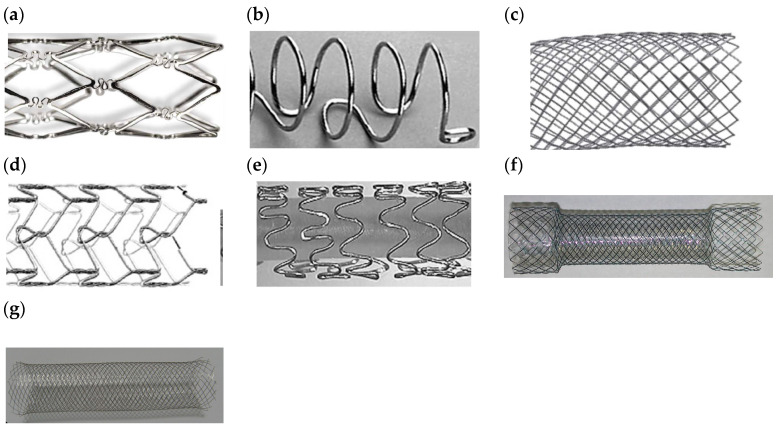
Designs of stents: (**a**) slotted tube, (**b**) coiled stent, (**c**) braided stent, (**d**) knitted stent, and (**e**) helical. Reproduced with permission [30]. Copyright 2022, Elsevier. (**f**) covered, (**g**) uncovered stents. Reproduced with permission [95]. Copyright 2010, Elsevier.

Generally, helical patterns are favored due to their high flexibility with few or no internal connection points, but this comes at the cost of lacking longitudinal support. Consequently, they may experience elongation or compression during delivery and deployment, leading to irregular cell sizes. On the other hand, designs with internal connecting points such as open cells and closed cells, compromise some flexibility in exchange for more excellent longitudinal stability and enhanced control over cell sizes [52]. A representative of the discussed designs is shown in Figure 9 and summarized in Table 4.

#### 4.2.3. The Effect of Geometry on the Final Properties of Stents and Hemodynamic Factors

Despite the generally favorable qualities of stents, complications can arise following their implantation. Restenosis and thrombus formation can primarily occur due to damage to the coronary endothelial cells caused by the stent. Drug-eluting stents, which release medications through the stent, can mitigate these complications. However, late stent thrombosis has the most significant clinical impact, leading to severe consequences, such as stroke and heart failure.

Research has indicated that stent struts alter blood-flow patterns, resulting in abnormal hemodynamics characterized by low velocities and, consequently, reduced wall shear stresses (WSSs). WSS is a valuable indicator for evaluating the hemodynamic performance of a stent design. Computational fluid dynamics (CFD) offers an efficient means to enhance stent designs by swiftly and cost-effectively analyzing various parameter configurations. For instance, numerous research groups have identified the height of the struts extending into the vessel lumen as a critical parameter influencing thrombus formation [96].

Various studies have shown that low WSS and high oscillatory shear index (OSI) can lead to restenosis. Therefore, it is essential to analyze these parameters together. One of the main factors influencing these parameters is the design of the stent [97]. Pant et al. revealed that the length of the struts and their alignment with the flow were the factors that most influenced hemodynamics. In another study led by Gori, open-cell stents and closed-cell stents were compared. Their results showed that closed cell stents were more adequate to avoid restenosis [97].

Stent strut thickness is another important issue with a great impact on hemodynamics. In research by Lannaccone et al., it was shown that stents with thinner struts and higher number of connectors could reduce both stent thrombosis and target lesion revascularization [98]. Stent strut thickness, affecting arterial wall injury at the time of implantation, as well as blood rheology after implantation, results in inflammatory responses at the target lesion, re-endothelization process, struts coverage, and neointima formation. Thinner struts result in lower inflammation response, which leads to less thrombogenicity, less neointimal hyperplasia, and a lower risk of target lesion revascularization [99].

## 5. Characteristics

NiTi, a biocompatible material with high corrosion resistance and damping properties, is favorable for biomedical devices. The unique behavior of shape memory effect and superelasticity of this alloy is a plus advantage that led to the advent of self-expandable stents [100]. In the following subsections, we elaborate on these characteristics, processing, and post-processing approaches to improve them.

### 5.1. Surface Characteristics

**Figure 10:**
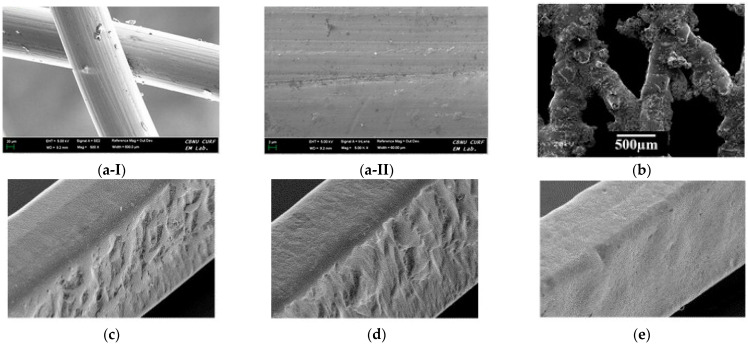
Surface characteristics of NiTi stents: bare NiTi stent struts at (**a-I**) low and (**a-II**) high magnification. Reprinted from open access journals [106]. (**b**) stent manufactured by LPBF. Reprinted from open access journals [107], (**c**) surface after mechanical polish, (**d**) after passivation, and (**e**) after electropolishing [108]. Copyright 2006, Elsevier.

**Figure 11:**
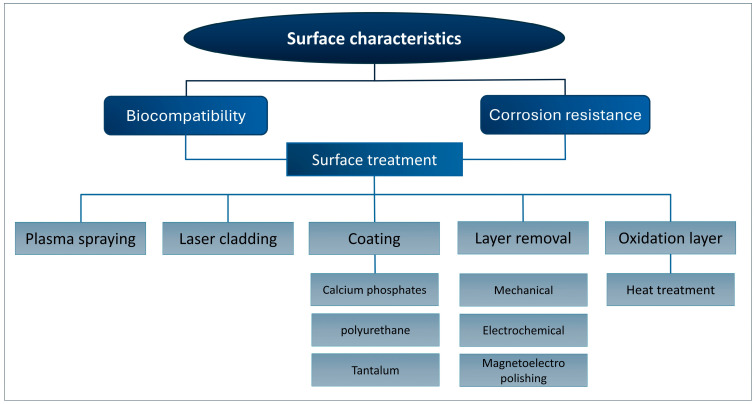
Different surface characteristics of stents. This illustration shows that biocompatibility and corrosion resistance are affected by the post-surface treatments.

Apart from the careful processing of NiTi to achieve optimal shape memory and superelasticity [101], in vitro studies show that biocompatibility and corrosion resistance depend on the surface characteristics of stents [102]. The surface morphology of the stent is directly related to the interaction between the stent with cells. Surface treatments such as electropolishing and laser treatment results in smoother finish surface that leads to less post placement complications such as thrombosis. A few examples of the stent surface are presented in Figure 10. The release of Ni ions due to the poor surface finish is one of the main concerns in NiTi stents. Therefore, surface treatment is a vital step to enhance the properties of nitinol to make it a compatible material for stents [46]. Another drawback of poor surface finish is the porosity and crack initiation susceptible regions that adversely affects the mechanical properties. Cardiovascular and artery stents require optimal surface roughness for smooth blood flowability and coating [103]. The surface produced by LPBF has high surface roughness of more than 10 μm, which can cause dimension deviation [104]. A smooth surface with optimal roughness lowers the risk of in-stent restenosis and thrombosis. Surface wettability, which is defined by how easily a liquid is spread on the surface, is reported to affect the biological response of nitinol. This characteristic is measured by the contact angle of a liquid droplet on the surface. A lower contact angle results in better cell attachment and reduces the risk of thrombosis [105]. As mentioned, surface properties are related to the biocompatibility and corrosion behavior of stents, as shown in Figure 11.

### 5.2. Corrosion Properties

Being a passivating material, nitinol has a corrosion resistance comparable to steel. The formation of a dense titanium oxide TiO_2_ film on the surface prevents extensive corrosion. Nevertheless, studies reported that this self-passivating nature of NiTi can be affected by factors such as the composition and microstructure of the alloy and the corrosive media. Therefore, several methods are used to improve the corrosion resistance of NiTi, such as laser cladding, surface coating, plasma spraying, and carbon layer deposition [109]. Feng et al. [110] investigated the effect of adding Ni on the corrosion resistance of NiTi coating on 316-stainless-steel substrates. Adding Ni created fine and uniform dendrites and improved the formation of the passive film. In a recent study, Yang et al. [109] coupled femtosecond and nanosecond lasers to create a dense multilayer structure on nitinol, isolating it from the corrosive medium and thus improving corrosion resistance. Surface oxidation resulting from the post-heat treatment of stents has been proven beneficial to the corrosion resistance of stents [111].

The release of Ni ions is a significant concern regarding NiTi stents. Improving surface quality by polishing and thermal oxidation can prevent the release of toxic Ni ions into the blood and tissues. Among these methods, electropolishing depicted better corrosion behavior than mechanical polishing, chemical polishing, and thermal oxidation [112]. Rokicki’s team [113] reported that the magneto-electropolishing process is more beneficial to corrosion resistance than electropolishing. Electropolishing can increase the amount of released Ni, whereas magneto-electropolishing can form a pure titanium oxide layer. Bae et al. [114] used salt heat treatment and investigated the resulting corrosion behavior. They reported that NiTi heat-treated in salt furnaces exhibits increased corrosion resistance compared to those in air furnaces. In another study, Say et al. [115] investigated two biocomposite coatings of silver on NiTi: hydroxyapatite/silver and bioglass/silver. Their analyses showed improved corrosion resistance for both coatings, with the hydroxyapatite/silver coating showing better corrosion resistance, a crack-free surface, and a higher adhesion strength of up to 79%. In a study by Mazumder et al. [116], the corrosion resistance of nitinol stents and its variation with different polymer-coating thicknesses was examined in an artificial physiological solution using a Potentiostat/Galvanostat and an electrochemical corrosion cell. The corrosion rate significantly reduced from 275 μm/year for the uncoated surface to below 13 μm/year with a 30 μm thick polyurethane coating.

### 5.3. Biocompatibility

Biocompatibility directly relates to the successful application of medical devices implemented in the body. The biocompatibility of nitinol stents is highly dependent on the corrosion resistance. NiTi generally does not provoke significant immune or inflammatory response from the human body, which is beneficial for its use in stents. This characteristic, known as the bioinert nature of NiTi, helps to reduce the rejection of stent by body and the post-stent-placement complications, such as thrombosis. Proper coating of the stent and increasing its corrosion resistance, as well as surface treatments, improve its bioinert nature [117].

NiTi is known as a compatible material due to the formation of a TiO_2_ layer on the surface that prevents the release of toxic Ni ions. The relatively high amount of Ni in nitinol stents can lead to toxicity and harmful reactions with body cells, resulting in inflammation and restenosis [118]. Therefore, surface treatments such as layer removal, coating, and oxidation play an important role in the interaction of the stent with the body. Several coating materials were analyzed to evaluate the biocompatibility of the stent. Coating NiTi with calcium phosphates is proven to be an effective way to enhance biocompatibility with cell tissues compared to mechanically removing a surface layer [119]. Rodrigues et al. [120] achieved improved biocompatibility of NiTi stent by covering it with polyurethane with successful follow-up monitoring of 6 months. Tantalum coating deposited on roughened NiTi strengthens biocompatibility and has proven beneficial to endothelial cell proliferation [121]. As discussed, surface oxidation has also proven helpful to the corrosion resistance and, thereby, biocompatibility of the stents.

Despite biocompatibility and corrosion resistance of NiTi, its insufficient antibacterial activity limits its cardiovascular applications. Coating of NiTi is reported to improve antibacterial properties of nitinol. Researchers have found out that incorporating antibacterial agents either as alloying elements or within a surface-modified layer can improve the antibacterial properties [122]. Ahmed et al. used biocomposite of AuNPs and a polymer coat on NiTi and reported improved antibacterial performance as well as improved corrosion resistance [123] [124]. The deposition of Ta in the presence of oxygen and argon gas resulted in the formation of Ta_2_O_5_ oxide layer on NiTi alloy and increased the antibacterial properties [125].

### 5.4. Wear Properties: Friction between Stent and Artery

Another important property of a material used for implants is its wear properties [126]. Interaction between objects depends on the materials, and it is essential to study the friction between body vessels and tissues with the inserted implants. Measuring the coefficient of friction and contact pressure, two important characteristics of this interaction, is mainly investigated numerically due to the challenges associated with experimental approaches [127]. Besides corrosion resistance and biocompatibility, surface treatment affects the wear properties. Ion implantation is a widely used method to improve the wear properties of NiTi shape memory alloy by forming an amorphous layer on the surface. The amount of improved wear resistance can vary based on the microstructure of the alloy and the used ions, such as N, C, and He [128]. In a study by Ma et al. [129], an ultrasonic nano-crystal surface-finish treatment of additively manufactured NiTi resulted in a hardened surface layer and, consequently, higher wear resistance.

### 5.5. Fatigue and Durability

Fracture is one of the main challenges in stent application, and it can cause injury to vessels. After one year of implantation, 50% of the fracture was reported in bending and twisting loading. Stents typically undergo 40 million cycles yearly, making fatigue and durability investigation highly important in stent fabrication [130]. NiTi self-expanding stents are used in peripheral arteries, such as superficial femoral arteries and renal arteries with complex functions of bending, stretching, twisting, and compression environment of blood vessels. The surface finish and design of the stent can affect its failure. A poor surface finish and the presence of porosity and cracks on the surface can lead to fatigue and failure. Electropolishing is used to improve the fatigue resistance of stents [131]. The design of the stent to better accommodate the movement of the body and changes in the arteries can hinder failure [132]. The strain can change from 3 to 10%, depending on factors such as patient blood pressure [133]. Therefore, the shape-memory effect and superelasticity of NiTi are strong reasons for this material to be used for stent fabrication. Pelton et al. [130] tested diamond-shaped NiTi samples for 10^7^ cycles and reported increased fatigue life at strains above 1.5%. There are limited studies on the fatigue fracture of NiTi [133], and numerical approaches are reported as promising approaches to predicting the fatigue lifetime of human arteries. Harvey et al. [134] used FEM to predict the fatigue performance of two types of NiTi stents, and their predictions indicate the direct influence of the design on the fatigue performance of the device. Their findings can be used to improve the design of stents, for example, considering the nonuniform distribution of strain.

### 5.6. Shape Memory and Superelasticity

**Figure 12:**
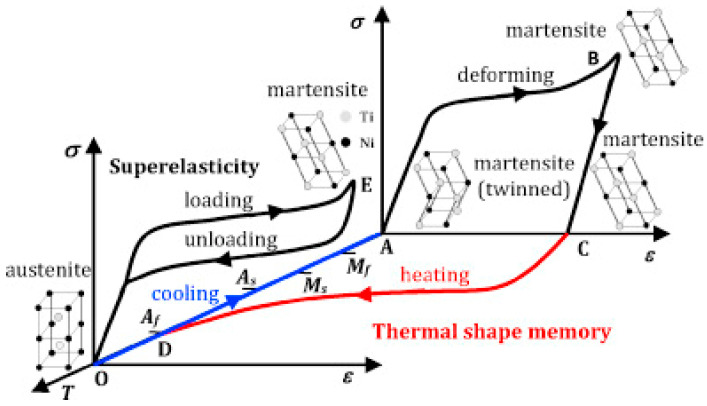
Stress–strain–temperature diagram of NiTi. Reproduced with permission [136]. Copyright 2013, Elsevier.

The shape memory and superelasticity of nitinol make it a suitable material for various applications. Temperature-induced phase transformation, known as the shape-memory effect, is mainly used in aerospace and actuators. Superelasticity, which is defined as phase transformation induced by stress, is mainly used in medical devices and body implants where the ambient temperature, i.e., body temperature, is stable [46]. A recent study by Finazzi et al. [135] investigated the superelasticity of NiTi stents produced by LPBF. Upon mechanical loading, conventional materials such as steel with limited elastic deformation of up to approximately 1%, are quite different from organic tissues in the human body [46]. Nitinol, on the other hand, can deform up to 8% strain. This is due to its unique features known as shape memory effect and superelasticity, based on the two main phases of martensite and austenite in NiTi, with different crystal structures and their reversible transformation [101]. As shown in the stress–strain curve of NiTi at body temperature in Figure 12, upon loading, NiTi has elastic behavior, and upon further loading, it changes its shape. This stress-induced phase transformation makes NiTi a suitable material for self-expanding stents.

## 6. Biocompatibility and Hemodynamics of Stents

Following stent implantation, the direct contact of the stent with blood steam can lead to various reactions, depending on the stent’s compatibility with blood, often referred to as hemocompatibility. These reactions may include inflammatory and allergic responses, thrombus formation, and in-stent restenosis (the recurrence of vessel narrowing). They are influenced by factors such as the stent’s material, the surface treatment, the structural shape and design, alterations in local hemodynamics, the method of deployment, and the extent of intrusion into the vessel wall.

The review by Liu [137] reported different mechanisms of platelet activation and aggregation, as well as the role of protein clotting factors in the thrombosis pathways, leading to in-stent thrombus formation and accumulation. Additionally, platelets and other contributory factors may trigger smooth muscle-cell activation and intimal hyperplasia, potentially leading to in-stent restenosis, as reviewed by Mitra and Agrawal [138].

The above-mentioned hemocompatibility can be assessed by various metrics, such as measuring the rate of hemolysis, the adhesion of platelets to the stent struts, the fibrinolysis rate, and the activation rate of certain coagulation factors [137]. The choice of metric and assessment may be conducted under different settings, such as in vivo, ex vivo, or in vitro conditions.

Over the decades, efforts have been made to enhance stent hemocompatibility and improve clinical outcomes. These improvements have involved implementing surface-modification techniques such as depositing a thin uniform coating, developing a stable passivation oxide layer, ion-beam processing for surface modification, and surface texturing, as suggested by Liu [137]. For instance, the application of surface coatings with inorganic materials, including titanium–oxide [139], titanium–nitride–oxide [140], and copper–titanium [141], has shown favorable effects on hemocompatibility. For further information on the biocompatibility and corrosion resistance of nickel–titanium in vascular stents, readers can refer to studies by Barras and Myers [142], Stoeckel et al. [46], Chakraborty et al. [143], and Maleckis et al. [144].

Furthermore, the recent reviews by Torii et al. [145] and Koźlik et al. [94] discussed the use of drug-eluting materials, which have been widely employed as either surface coatings or as the sole stent material. The first generation of drug-eluting stents encountered significant issues that resulted in adverse cardiovascular reactions over time. However, the more recent generations have demonstrated improved efficacy and safety.

An alternative approach involves fabricating laser-induced periodic surface structures (LIPSSs) on vascular stents. The adaptive and precise method of laser surface engineering offers opportunities for treating stent materials and creating various texture patterns. This approach also allows for modifications to the surface chemistry through nitridation, oxidation, and coatings, as suggested by Saqib et al. [146] and Grossmann et al. [147]. These treatments may help reduce platelet adhesion and thus control corrosion behavior and degradation, as reviewed by Dong et al. [148]. The laser-texturing process applied to commercially available stents has also been investigated by this team and has shown promise in stent treatment.

From a hemodynamics standpoint, several metrics, such as wall shear stress, relative residence time (RRT), oscillatory shear index, flow separation, and reattachment, have been suggested to be linked to the stent inflammatory responses, localized in-stent thrombosis, in-stent restenosis, and vascular remodeling. For example, in vitro experiments and CFD simulations have demonstrated that non-streamlined stent struts deployed at the arterial surface in contact with the bloodstream would cause localized proximal and distal flow recirculation zones, as indicated by studies of Jiménez and Davies [149], Jiménez et al. [150], and Nguyen et al. [151].

This flow condition induces a prolonged blood residence time near the vessel wall, often leading to a low wall shear stress region. The WSS is commonly used as a critical hemodynamic biomarker (perhaps the most widely used hemodynamic metric). A low-shear rate may be linked to a thrombosis pathway and a reduced likelihood of endothelialization, as indicated by Sprague et al. [152] and Antoniadis et al. [153].

However, thrombus formation is a complex and multifactored process that may occur through a high-shear pathway, as demonstrated by Casa et al. [154] and Casa and Ku [155]. Overall hemodynamics plays an important role in this thrombosis pathway, involving von Willebrand factor (vWF) binding, platelet adhesion, platelet activation, and thus rapid thrombus growth.

In general, designing and fabricating vascular stents that minimally alter local hemodynamics while maintaining a good compliance match between the stent structure and the vessel wall can help reduce the risk of thrombosis and restenosis. For further reading, see the studies by Berry et al. [156], Hoi et al. [157], Brindise et al. [158], Xu et al. [159], and Williamson et al. [160].

It is important to note that the mentioned biocompatibility and biofluid mechanics apply specifically to vascular stents. While nitinol has the important superelasticity and shape-memory properties that are favorable for the recanalization of obstructed airways, for tracheal and airway stents the reader is referred to reviews by Sabath and Casal [161] and Tian et al. [162].

## 7. Performance

### 7.1. Patency Rate

Over 75% of stents do not remain open naturally within a year, requiring extra interventions due to the narrowing caused by excessive cell growth on and near the stent, known as restenosis. Therefore, it is crucial to pinpoint the factors linked to this recurrence after stent insertion [163]. According to the Society of Vascular Surgery standard, the primary patency rate refers to the absence of stent blockage without requiring extra surgical or endovascular interventions. On the other hand, the secondary patency rate is the percentage of patients who sustain the patency of the stent following an additional surgical or endovascular procedure conducted after the stent has become blocked [164]. The greater the size of both the vein and the stent, the more favorable the patency rate becomes. Research has demonstrated that the dimensions of the vessel and stent significantly impact the volume of blood flow through the stent after the procedure and the rate of restenosis [163].

To estimate the performance regarding the main clinical effectiveness measure, vessel patency, a literature review conducted by Rocha-Singh and colleagues examined peer-reviewed publications spanning over 15 years. In their review, they identified five randomized trials where 203 patients in the control group were randomly assigned to undergo percutaneous transluminal angioplasty (PTA) with duplex ultrasound (DUS) assessment of vessel patency up to 12 months. Out of the 203 patients randomized to receive PTA, 191 patients were adequately followed up to evaluate patency at the 12-month mark. The primary patency rate recorded at 12 months was 37%. The researchers combined the findings from this literature review with the results from the control group undergoing PTA provided by device manufacturers. This manufacturer-provided data indicated a patency rate of 28%. Combining these two sources, they arrived at an overall PTA patency rate of 33%. It is worth noting that the 12-month PTA patency rates from these two sources, involving a total of 277 patients, were quite similar (37% and 28%) [165].

Murphy et al. achieved all the pre-established performance targets, covering the main effectiveness and safety criteria. Their results demonstrate a noteworthy primary patency rate of almost 90%, followed by similar rates for primary-assisted and secondary-assisted patency, and a minimal occurrence of only 2% major adverse events (MAEs) recorded within a 30-day timeframe [166].

Grogan presented an innovative technique to replicate the corrosion process in Absorbable Metal Stents (AMSs) as part of a study examining the impact of corrosion on the mechanical characteristics of different stent designs. This corrosion model is combined with an optimization strategy to pinpoint the specific features of AMS that provide the best resistance to corrosion within the body [167].

To further evaluate how the Zilver PTX stent performs in real-world situations, researchers conducted a prospective multicenter study referred to as the Zilver PTX for the femoral artery and proximal popliteal artery (ZEPHYR) registry [168].

Grogan and collaborators conducted a computational investigation to study how the choice of materials affects the performance of coronary stents. They considered various materials, including magnesium alloy, iron, steel, and cobalt–chromium stents. Their evaluation incorporated both generic and material-specific geometric models. They assessed stent performance through simulated bench tests, using established modeling techniques in the current literature or those that provided predictions readily comparable to in vitro experimental outcomes [169].

The need to enhance the thrombogenic performance of cardiovascular devices that circulate blood, such as prosthetic heart valves (PHVs) and ventricular assist devices (VADs), is emphasized due to the requirement for lifelong anticoagulation therapy with these devices. It is important to note that even with such therapy, the risk of thromboembolic complications is not entirely eliminated [170].

### 7.2. FDA Approvals

The initial step toward achieving an optimal outcome involves precise placement, after which the stent’s properties, such as radial strength and flexibility, become significant. Consequently, innovations in the field of stents have concentrated on developing smoother and more accurate stent delivery systems.

As an illustration, in August 2015, Boston Scientific Corporation announced that it had received FDA approval for the Innova vascular self-expanding stent made from nitinol. This stent was equipped with an advanced delivery system designed to treat peripheral artery disease in the superficial femoral artery or proximal popliteal artery. It came in various diameters, ranging from 5 to 8 mm and lengths spanning from 20 to 200 mm. The Innova platform featured a hybrid cell structure that included open cells along the stent’s body and closed cells at each end, ensuring consistent and precise deployment. Furthermore, the Innova stent system was engineered with a triaxial delivery system to ensure accurate and predictable stent placement, as well as uniform deployment, according to the company’s statement [171].

Deep venous obstruction occurs when the veins within the deep venous system become blocked, compressed, or obstructed, leading to restricted blood flow toward the heart. If left untreated, individuals can experience discomfort and pain in their legs, significantly limiting their mobility and overall quality of life. Symptoms of this condition include leg swelling, skin changes, leg ulcers, and various forms of leg pain. In more severe cases, complications such as blood clots traveling to the lungs (known as pulmonary embolism), the formation of deep vein thrombosis (a clot in the leg), or the development of fibrotic tissue and scarring due to chronic post-thrombotic syndrome can occur. As of October 2020, the U.S. Food and Drug Administration (FDA) has granted clearance for the Medtronic Abre venous self-expanding stent system. This clearance was based on a 12-month study demonstrating an overall primary patency rate of 88%, with no stent fractures or migrations [172]. This type of stent is specifically indicated for use in the iliofemoral veins of patients diagnosed with symptomatic iliofemoral venous outflow obstruction, commonly called deep venous obstruction.

In July 2022, BIOTRONIK announced its receipt of FDA approval for the innovative Pulsar^®^-18 T3 peripheral self-expanding stent system, designed to enhance the implantation process for endovascular treatments. The redesigned Pulsar-18 T3 stent system introduces a user-friendly, wheel-operated handle that allows physicians to release the stent with one hand, improving their control during deployment. Clinical data underscore the long-term safety and effectiveness of the Pulsar stent, with a remarkable 89.3% freedom from target lesion revascularization rate and no major target limb amputations reported at the 24-month mark. The Pulsar-18 T3 stent system is intended for use in patients with symptomatic de novo, restenosis, or occlusive lesions in the superficial femoral or proximal popliteal arteries. It is suitable for reference vessel diameters ranging from 3.0 to 6.0 mm and total lesion lengths of up to 190 mm [173].

The Abre venous stent is designed for utilization in the iliofemoral veins. It comprises the stent itself and an associated delivery system. The stent component is constructed using an open-cell nitinol self-expanding design. Notably, it incorporates three offset connection points that intentionally spiral down the stent, aiming to enhance flexibility while reducing the likelihood of stent kinking or instability during deployment. Moreover, the length and thickness of the stent struts are adjusted based on the stent’s diameter to ensure consistent stent strength across various stent diameters [172].

## 8. Modeling/Simulation Studies

Coronary stents are at the forefront of advancements exploring factors that affect their effectiveness and clinical outcomes. These factors include design considerations, the properties of biomaterials in terms of the mechanics and chemistry of different coating options, drug release characteristics, and the crucial role played by cardiologists in treating specific coronary lesions. The successful deployment of stents relies on achieving a balance of attributes to ensure long-term implantation without rejection. These attributes include performance, flexibility, strong radial strength, durability, and resistance to corrosion [174].

When evaluating and optimizing stent performance and efficacy, it is essential to consider the range of commercially available options, such as different geometric designs, materials used, coatings applied, and pharmaceutical agents employed. While clinical experiments and laboratory studies provide insights into arteries response to stent implantation, they are often costly and time-consuming. In contrast, computer-based numerical simulations offer advantages in terms of flexibility control over variables involved in the analysis process and cost effectiveness. Computational modeling provides a framework for gaining insights into factors that influence successful stenting outcomes. It serves as a tool for exploring hypothetical scenarios using techniques such as finite element analysis (FEA) and computer-driven methodologies.

Recent advancements in both hardware and software have significantly expanded the scope and predictive precision of computational simulations related to stent deployment. These improvements have substantially reduced the time required for tasks such as geometry creation, pre-processing, numerical solution, and post-processing. Notably, finite element analysis and computational fluid dynamics stand as the principal simulation disciplines, complemented by the utilization of artificial intelligence (AI). These computational approaches facilitate assessing, predicting, and optimizing process–structure–properties–performance (PSPP) relationships for stents [175], even before their production.

### 8.1. Mathematical Modeling of Stents

The constitutive model proposed by Fereidoonnezhad et al. [176] has been employed to characterize the behavior of arterial layers. This model, characterized as inelastic, comprehensively incorporates hyperelasticity, stress softening, and permanent deformation characteristics of arterial tissues, particularly post-stent insertion. Within this model, local deformation is distinguished into volumetric and isochoric components. As such, the deformation gradient is defined as $F=J^{1/3}\bar{F}$, with J=det⁡F, representing the volume ratio, and F denoting the modified deviatoric deformation gradient. Consequently, C=FTF signifies the right Cauchy–Green deformation tensor [177,178], albeit in its modified form.

The arterial material is assumed to exhibit nearly incompressible behavior, leading to the formulation of the strain energy density function Ψ as $\Psi \left(C,a_{04},a_{06},\eta_{s,4},\eta_{s,6},\eta_{in}\right).$ Additionally, $\Psi_{vol}\left(J\right)$ is expressed as $\frac{1}{D}(\frac{J^{2}-1}{2}-\ln J)$, where D serves as a material parameter, and $\Psi_{vol}$ and $\bar{\Psi }$ represent the volumetric and isochoric energy, respectively. Notably, the damage phenomenon is exclusively applied to the isochoric energy component, encompassing stored energy within the reference configuration, fiber behavior, and associated damage parameters.

$\bar{\Psi }={\bar{\Psi }}_{m}^{0}\left({\bar{I}}_{1}\right)+\sum_{i=4,6}\left[\eta_{s,i}{\bar{\Psi }}_{f,i}^{0}\left({\bar{I}}_{1},{\bar{I}}_{i}\right)+\phi_{i}\left(\eta_{s,i}\right)\right]-[\left(1-\eta_{in}\right)N\left(C\right)+\phi_{in}(\eta_{in})]$ [179,180]. 

In this intricate formulation, denoted by mathematical symbols, various parameters and functions are crucial for characterizing the behavior of arterial tissue and its interaction with stents. To elaborate in a structured and professional manner, two families of fibers embedded within arterial tissue, identified by angles θ concerning the circumferential direction, are represented by $a_{0i}$, i=4,6, denoting their respective directions. These fibers play a pivotal role in the tissue’s mechanical properties.

N encompasses the inelastic energy, while $\phi_{i}$ and $\phi_{in}$ are dissipation functions. Damage variables, $\eta_{s,i}$ and $\eta_{in}$, are introduced to account for stress softening and permanent deformation behaviors. Additionally, key invariants are defined:

$I_{1}=trC$, $I_{4}=a_{04}$. ${Ca}_{04},I_{6}=a_{06}.Ca_{06}$, ${\bar{I}}_{1}=J^{-2/3}$, ${{\bar{I}}_{4}=J^{-2/3}I}_{4}$, and ${{\bar{I}}_{6}=J^{-2/3}I}_{6}$ [180].

The stored strain energy functions in the ground matrix (${\bar{\Psi }}_{iso}$) and fibers (${\bar{\Psi }}_{f,i}^{0}$) are expressed as follows:

${\bar{\Psi }}_{m}^{0}\left({\bar{I}}_{1}\right)=µ({\bar{I}}_{1}-3)$, ${\bar{\Psi }}_{f,i}^{0}\left({\bar{I}}_{1},{\bar{I}}_{i}\right)=\frac{k_{1}}{2k_{2}}[\exp\left(k_{2}{\bar{E}}^{2}i\right)-1]$, and ${\bar{E}}_{i}=k{\bar{I}}_{1}+\left(1-3k\right){\bar{I}}_{i}-1$ [176].

Here, µ, $k_{1}$ and $k_{2}$ represent critical material parameters, with k confined to the interval [0,1/3] signifying the dispersion parameter.

The inelastic energy and damage variables are presented as follows:

$N\left(C\right)=µ\left({\bar{I}}_{1}-3\right)+\frac{k_{1}}{2k_{2}}\sum_{i=4,6}\left[\exp\left(k_{2}{\bar{E}}_{i}\right)-1\right]$, ${\bar{E}}_{i}=k{\bar{I}}_{1}+\left(1*3k\right){\bar{I}}_{i}-1,\eta_{s,i}=1-\frac{1}{r_{1}}\mathrm{erf}\left[\frac{1}{m_{1}}\left({\bar{\Psi }}_{f,i}^{max}-{\bar{\Psi }}_{f,i}^{0}\right)\right]$, $\eta_{in}=\tanh\left[{\left(\frac{{\bar{\Psi }}^{0}}{{\bar{\Psi }}^{max}}\right)}^{m_{2}}\right]/tanh(1)$ [180].

In these equations, $µ,k_{2},m_{1},k$, and $m_{2}$ serve as material parameters that describe anisotropic damage in collagen fibers. Furthermore, ${\bar{\Psi }}_{f,i}^{max}$ and ${\bar{\Psi }}^{max}$ represent the maximum strain energy of fiber family i and the tissue, respectively. These values are derived from the deformation history, depending on the modified right Cauchy–Green tensor at the peak deformation of the loading history. To implement this constitutive model, it is implicitly discretized and incorporated into a user-defined material subroutine (UMAT), making it compatible with finite element method (FEM) software [176,180].

### 8.2. Simulated Stents Undergone Finite Element Modeling (FEM) and Computational Fluid Dynamic (CFD)

Rapid development of medical imaging techniques and computational methods, such as the finite element method, allows investigation of the impact of stenting on vessel walls. Many investigators have used finite element analysis to test different stent designs for their low cost compared to testing different prototypes [181]. This method can study other mechanical properties of stents, such as flexibility and stent-tissue interactions.

Most importantly, the FEM method serves as a valuable preclinical tool to analyze existing stent designs and to develop new stent designs. It is hypothesized that vascular injury is the stimulus for restenosis formation. Restenosis is thus linked to stent design. The depth of penetration of the stent wires is one of the factors that determines the degree of restenosis [182].

Finite element models are thus a central part of stent improvement and development [178]. Geometrical parameters and material properties can be considered. Model parameters include displacements, radial stiffness, bending flexibility, stresses, and strains. Also, interaction with the artery wall may be considered. The FEM can provide a quantitative assessment of the relation between the complex mechanical features of a given carotid stent design and a given patient-specific carotid artery anatomy. This could be useful for procedure standardization. It may be thus beneficial to develop FEM models of realistic vessel geometries that will allow choosing a stent design based on the stenosis geometry of each individual. FEM can also be used for simulations of manipulation with a catheter. It can help in the preoperative choice of stent parameters by modifying its properties according to real 3D images from magnetic resonance angiography [183].

In summary, FEM is used to investigate several aspects of coronary stenting including the evaluation of the interventional techniques, the impact of plaque composition on vessel wall stress, and carotid artery stenting. The performance of different self-expanding stent designs in the carotid artery (CA) can be evaluated using FEM. The FEA is thus a powerful technique to investigate the impact of stenting on the vessel wall. Using FEA to analyze carotid artery stenting (CAS) as a procedure planning tool to support clinical practice is challenging. A realistic simulation environment for CAS will allow pre-operative and post-operative information comparison.

Specifically, FEM methods allow to model the different properties and design considerations for self-expanding nitinol stents [43]. The most unusual property of nitinol alloys is stress hysteresis. Various designs have been identified and can be modeled, including wire-based, sheet-based, and tube-based stent designs. In conclusion, FEM allows us to explore the effects of new stent designs on the stenting procedure.

The FEM method was used to determine the effects of material properties on the mechanical performance of nitinol stent [184]. Open-cell Z-shaped rings were investigated. Abaqus software v. 6.10 was used to conduct the analysis along with the hypermesh software v. 6.0 to allow for the complex geometry and wire section of the NiTi stents. MSc Nastran software was used to investigate how different stent designs affect the stress in the vascular wall and showed that stress predictions can be correlated with in-stent restenosis [182]. It was possible to determine the effect of stent placement on vessel wall stresses and stent-induced vascular injury.

COMSOL was used to predict the deformation of a stent in the artery, during artery deformation and when pushed from the catheter [183].

The exact mechanical characteristics of a stent were determined using ABAQUS to model tubular and coil-type balloon stents [185].

Abaqus was also used to generate a patient-specific CA model. Self-expanding laser-cut open-cell, laser-cut closed-cell, and braided closed-cell stent designs were modeled in different sizes and configurations and used to simulate deployment in the CA model [186]. Vessel stresses were estimated, lumen gain, and vessel straightening were evaluated by comparing the pre- and post-stenting vessel geometry.

ABAQUS was further used [187] to investigate the impact of carotid stent on carotid anatomy. A patient-specific model of CA was generated, which includes plaque. Two constitutive models for the vascular tissues were used to simulate the wall tensional state, the lumen gain, and vessel straightening. An open cell self-expanding nitinol stent with a straight configuration was modeled. The simulation consisted of the catheter being bent and crimped, then stent deployment allowing the stent to expand against the vessel wall. Plaque components need to be included in the model including lipid pool, necrotic core, calcific nodule, and fibrous cap. This requires high resolution images. Using CTA, healthy wall thickness cannot be identified, and plaque characterization requires special reconstruction algorithms. Also, ABAQUS assumes the contact between bodies to be frictionless. In order to determine the tangential component of the contact between stent and vessel, the friction coefficient of self-expanding stents needs to be determined. The challenge of including patient-specific prestress needs to be addressed. Prestress refers to the loading caused by the blood pressure.

Advancements in medical computational techniques and imaging technologies have ushered in a transformative era for investigating stent effects on vessel walls [188]. Notably, computational fluid dynamics and finite element analysis have emerged as pivotal tools for assessing hemodynamic factors and therapeutic outcomes pertaining to arteries and stents. Previous studies had a prevalent reliance on rigid 3D vessel geometries and non-deformable stent models. However, clinical observations have underscored the significance of artery deformation, particularly in regions with varying parent vessel curvature. This deformation leads to diverse flow patterns and hemodynamic conditions [189,190]. In the pursuit of heightened prediction accuracy and the mitigation of aneurysm risks during stent-expanding and balloon procedures, the finite element method has assumed a prominent role as a cost-effective pre-processing technique. It enables concurrent simulations of the artery and stent, thus offering a comprehensive approach to enhancing our understanding of these complex interactions.

Arteries undergo a dynamic adaptation process known as growth and remodeling (G&R), which involves producing or removing structural proteins like collagen to restore biomechanical equilibrium. Understanding this adaptation in the context of stent implantation is of paramount importance, as it can influence the progression of arteries toward unstable pathological conditions, such as aneurysms, with profound implications for patient health and survival. To address this critical need, a cost-effective, open-source finite-element 2D axisymmetric shell model of the arterial wall has been developed. This model is a valuable tool for predicting arterial adaptation following stent implantation and comprehending the consequences of oversizing on post-surgical outcomes [191]. It is worth noting that stent implantation can trigger diverse responses in both aneurysmatic and healthy arteries, with the latter often exhibiting more intricate and unstable growth and remodeling dynamics.

In the realm of simulating the mechanical behavior of stents during thrombectomy procedures, finite element analysis has emerged as a precious tool. Among FEA methods, Abaqus is a widely adopted choice for bio application simulations. Remarkably, researchers have harnessed the power of FEA, employing the Abaqus 6.14/Explicit solver, to conduct in-depth analyses of stent deployment and degradation within zinc alloys [192] and cobalt–chromium (CoCr) alloys [193].

**Figure 13:**
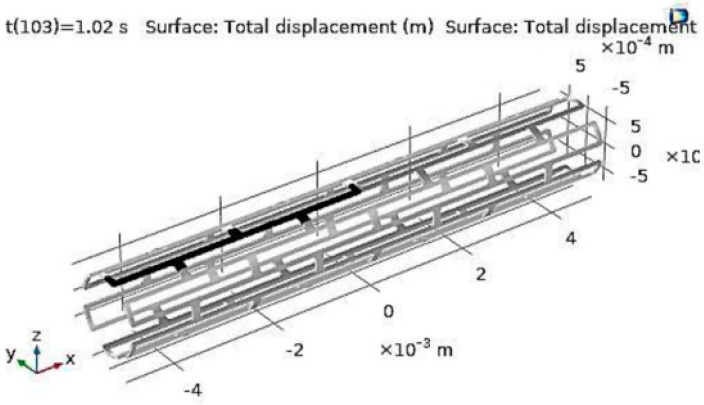
A finite element model utilizing the COMSOL Multiphysics^®^ numerical software has been constructed for the Palmaz Schatz stent, focusing on analyzing one twenty-fourth of the stent’s geometry. Reproduced with permission [196]. Copyright 2021, Elsevier.

To simulate the mechanical response of the biocompatible stent under internal inflation, an established finite element method approach involves the use of the COMSOL Multiphysics^®^ numerical software tool. The FEM simulations take into account a range of biocompatible materials, including Pure Mg [194], PLLA [195], nitinol [196], and 316L stainless steel [178]. The stent is represented with a non-uniform deformation, employing the Palmaz Schatz stent model and incorporating considerations for the shortening effect, as depicted in Figure 13.

**Figure 14:**
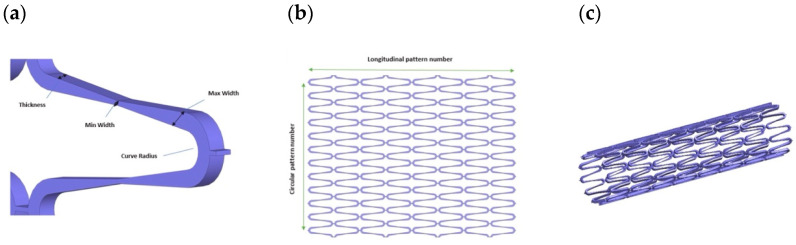
Stent size variation: (**a**) unit cell, (**b**) 2D pattern of cell, and (**c**) stent. Reproduced with permission [197]. Copyright 2020, Elsevier.

These simulations are not limited to static assessments but encompass dynamic changes in corrosion processes and material properties. Moreover, they consider critical factors such as pitting corrosion and stress corrosion, making them exceptionally comprehensive in their scope. In this context, key parameters, including amplitude, strut width, crown radius, and thickness (as depicted in Figure 14, are meticulously incorporated to facilitate a thorough understanding of stent behavior and performance.

### 8.3. Stents’ Process–Structure–Properties–Performance

To address the critical challenges associated with reducing mortality and morbidity in stenosis, thrombosis, atherosclerosis, and embolization involving stents, extensive computational modeling research has been undertaken. These studies delve into the intricate interplay between physiological variables in the artery and electrical variables. In particular, computational fluid dynamics has been employed as a valuable tool for dissecting the hemodynamic aspects of arterial behavior.

One noteworthy research endeavor led by Muhammad et al. [198] harnessed a lumped parameter mathematical model within a CFD framework to comprehensively explore the hemodynamic characteristics resulting from artery stenting. Parameters such as wall shear stress (WSS or $\tau_{w}$), time-averaged WSS (TAWSS), and wall shear stress gradient (WSSG) were analyzed. Additionally, the research aimed to optimize arterial conditions and minimize adverse effects, particularly the restenosis rate following stenting. The essential formulas involved in these analyses include $\tau_{w}=µ\frac{\partial u}{\partial r}|r=R$, where µ represents dynamic viscosity, u denotes blood flow velocity, and r signifies the radial distance to the center of the blood, with a radius of R; moreover, $TAWSS=\frac{1}{T}\int_{0}^{T}\tau_{w}dt$, where T signifies time. Furthermore, the Oscillating Shear Index (OSI), a nondimensional scalar used to assess the oscillatory nature of blood flows, is defined as follows:

$Oscillating\;Shear\;Index\left(OSI\right)=\frac{1}{2}(1-\frac{\int_{0}^{T}\tau_{w}dt}{\int_{0}^{T}\tau_{w}dt})$, [178].

Providing valuable insights into flow characteristics. The research findings indicated that low $\tau_{w}$ (<0.5 Pa), low TAWSS (<0.5 Pa), and high OSI (>0.1) were correlated with cellular proliferation, intimal thickening, and inflammation. These thresholds played a pivotal role in evaluating the impact of stenting [198,199].

Moreover, the phenomenon of “dogboning,” characterized by the expansion of both ends of a stent larger than its center during self-expansion, has substantial implications for thrombosis and hyperplasia. The equation used for assessing the dogboning effect is as follows:

$$Dogboning=\frac{D_{e}-D_{m}}{D_{m}}\times \%100,$$

where $D_{e}$ represents the mean diameter at the two ends, and $D_{m}$ denotes the mean diameter at the middle cross-section of the post-expanded stent. Stent recoiling, as another criterion to assess stenting performance, is a phenomenon associated with the elastic–plastic deformation of the stent and the loading pressure applied by the expanded artery, and it is defined as follows:

$$Recoiling=\frac{D_{max}-D_{unload}}{D_{unload}}\times \%100,$$

with $D_{max}$ and $D_{unload}$ representing the mean diameters of the middle cross-section of the stent during the holding and unloading phases, respectively. Stent foreshortening, which quantifies the difference between the desired and actual stent lengths after deployment, is described as follows:

$$Foreshortening=\frac{L_{0}-L_{load}}{L_{0}},$$

where $L_{0}$ is the original length of the stent, and $L_{load}$ is the deformed length of the stent [200].

Recent research has demonstrated the effective support provided by stents within vessels [193], resulting in a treatment efficacy exceeding 60%. Notably, adjusting the corrosion rate based on the number of exposed surfaces and reducing the stress threshold in response to the corrosion status has accelerated stent degradation by 26% and 25%, respectively, compared to the uniform degradation model. Additionally, stent amplitude and width variations significantly impact radial recoil and the pressure required for stent expansion. Increasing amplitude and width leads to higher radial recoil and expansion pressure, while decreasing them enhances the radial strength, dogboning ratio, and corresponding expansion pressure and foreshortening.

**Figure 15:**
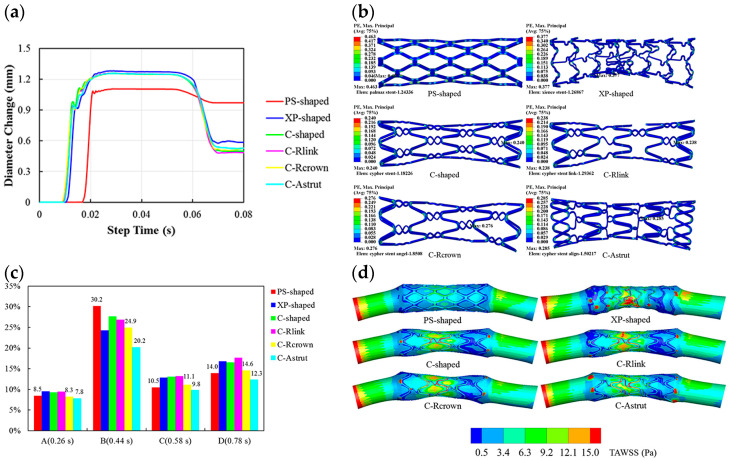
(**a**) A thorough investigation into post-processing analyses has been undertaken for four specific types of expandable stents, namely PLLA, nitinol, stainless steel (SS), and pure Mg, (**b**) along with an assessment covering six distinct geometrical variations in these stents. (**c**) These analyses encompass the evaluation of area percentages via histograms, with a primary focus on instances of adverse low WSS (<0.5 Pa) at four critical time points during a cardiac cycle. (**d**) Contour maps illustrate the distribution of time-averaged wall shear stress (TAWSS) on the lumen wall. Reproduced with permission [199]. Copyright 2019, Frontiers.

The effects of dogboning, foreshortening, and control over internal structure are simulated to optimize geometric parameters for peak performance. Consequently, the results highlight the maximum von Mises stress for nitinol (800 MPa) and minium (30 MPa) in pure Mg, along with an effective plastic strain for pure Mg (0.25). Furthermore, non-uniform deformation values for dogboning and foreshortening are tabulated for various bio-compatible materials. Moreover, a two-level elastoviscoplastic model has been developed, allowing direct control over the internal structure of the material, including parameters like grain orientation and grain size in a polycrystal [196]. Figure 15 shows the effectiveness of PSPP on more commonly used materials in stenting process.

Mejia et al. [201] introduced a comprehensive set of metrics centered on the statistical moments derived from the wall shear stress (WSS) distribution to evaluate the hemodynamic efficacy of a stent post-implantation. These metrics have exhibited promise in accurately assessing the performance of various stent strut profiles. Furthermore, their study revealed that ensuring appropriate strut apposition can significantly enhance stent hemodynamic performance. This underscores the potential for design modifications to enhance a stent’s hemodynamic capabilities. Numerous investigations have explored the influence of stent design on WSS distribution [202].

**Figure 16:**
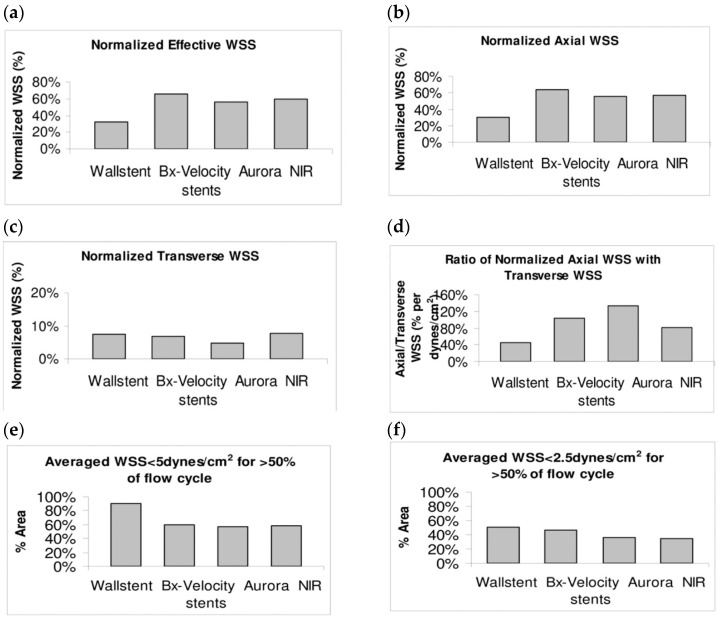
(**a**) Normalized effective wall shear stress (WSS), (**b**) normalized average axial WSS, (**c**) normalized average transverse WSS, and (**d**) ratio of normalized axial WSS to transverse WSS plotted for the various stent design types. Additionally, the percentage area of the region between struts with averaged low WSS ((**e**) <5 dynes/cm^2^ and (**f**) <2.5 dynes/cm^2^) for more than 50% of the flow cycle in the different stent design types. Reproduced with permission [204]. Copyright 2009, The American Society of Mechanical Engineers.

It is worth noting that prior research [201] primarily focused on individual strut profiles, lacking the capability to quantify the pivotal role played by the arrangement of struts within the stent’s unit cell. To comprehensively assess the process–structure–properties–performance relationship of a vascular stent, with a particular emphasis on its hemodynamic performance within a tubular geometry defined by the unit cell topology, Prithipaul et al. [203] developed a comprehensive model. This model incorporates structural metrics encompassing foreshortening, elastic recoil, and radial stiffness. Concurrently, hemodynamic performance is quantified through a wall shear stress index, which gauges the impact of in-stent restenosis. This assessment is based on a representative volume element (RVE) modeling approach. The findings of this study highlight the distinct structural and hemodynamic characteristics associated with each cell topology. This information can be instrumental in comparing and selecting stent geometries that strike an optimal balance in meeting clinical requirements. For a more detailed exploration of all five geometrical structures, please refer to Figure 16.

The simulated assessments, encompassing radial force and kink deformation evaluations, have revealed a robust alignment with empirical data. This alignment is particularly conspicuous in coated braided stents, where braid angles and cover thicknesses vary. The congruence between simulation results and empirical findings has been comprehensively elaborated upon in the research conducted by McKenna et al. [205]. Their exhaustive investigation delves deeply into the parameter–functional performance of stents, providing invaluable insights. The study conducted by McKenna and their collaborators underscores the pivotal role played by the braid angle as a governing parameter influencing the radial and kink performance of both bare-metal and covered wire-braided stents. Furthermore, their research emphasizes the substantial enhancement in radial responsiveness achieved by integrating a polymeric cover onto a wire-braided stent, particularly in scenarios involving thicker and more rigid covering systems.

**Figure 17:**
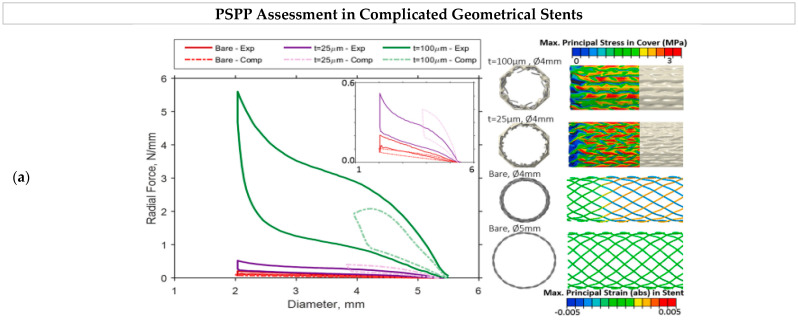

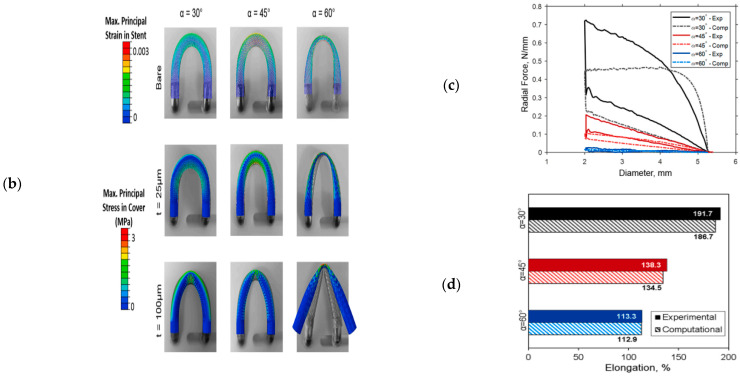
(**a**) This section presents a comprehensive comparative analysis of radial-force responses, drawing from both experimental and computational data, for wire-braided stents with braid angles of α = 45°. (**b**) Juxtapose computational bending deformations. (**c**) Comparison of experimental and computational data. (**d**) Stent elongation at 2.4 mm. Reproduced with permission [205]. Copyright 2021, Elsevier.

The most recent research provides further validation of these findings, reinforcing the precision of the computational model in forecasting radial-force patterns in braided stents with diverse braid angles and cover thicknesses, as visually illustrated in Figure 17**.** Upon meticulous scrutiny of the projected radial response of uncoated braided stents, it becomes evident that the model effectively captures the escalating radial reactivity as the braid angle decreases. While a slight underestimation of radial force at full crimp is noted, a phenomenon documented in comparable models [205], the overarching agreement is satisfactorily established.

In the crimped deformation analysis, the computational model consistently delivers precise forecasts of stent elongation at a 2.4 mm crimped profile. This alignment closely mirrors the empirically observed crimped profile following deployment within a glass tube, as visually exemplified in Figure 17d. In examining various cover thicknesses, specifically t = 25 μm and t = 100 μm, the model replicates the loading and unloading radial force profiles up to a crimped diameter of 3.95 mm. Furthermore, it offers valuable insights into the relative influence of cover thickness compared to experimental data.

It is worth highlighting that the model does disclose certain disparities. Precisely at a crimped diameter of 4.5 mm, the model exhibits a 64% overestimation in radial resistive force for braided stents with a cover thickness of t = 25 μm and a −19% underestimation for those with a cover thickness of t = 100 μm. Likewise, the chronic outward force at 4.5 mm is underestimated for braided stents with cover thicknesses of both t = 25 μm (−16%) and t = 100 μm (−6%). These variations can be partially ascribed to the intricate deformations within the cover material observed in crimped braided stents, where substantial folding and self-contact phenomena manifest.

Figure 17 serves as a comprehensive reference, providing an in-depth analysis of the anticipated kink performance for both bare-metal and covered wire-braided stents. This analysis includes direct comparisons with experimentally observed deformations [206]. In a broader context, the study underscores the diminishing kink resistance of braided-stent configurations as the braid angle and cover thickness increase. Impressively, these predictions closely align with experimental observations, effectively characterizing the buckling behavior displayed by stents featuring a braid angle of α = 60° and a cover thickness of t = 100 μm.

In more recent experimental studies, the investigation has shifted toward evaluating the fracture risk of nitinol peripheral stents under loading conditions that mimic the biomechanical deformations experienced by femoropopliteal stents during leg movements. Meoli et al. [207] leveraged computational modeling in their in vitro studies to enhance understanding of nitinol stent fatigue behavior. Their research specifically emphasized the role of the stent–wall interaction. In this context, two peripheral stent models underwent axial compression and bending, both in isolation within the fully expanded configuration and after deployment in a silicone tube designed to simulate the presence of an artery. This approach yielded distinct fatigue-behavior patterns, evident in terms of constant-life diagrams and strain distribution within the stents.

### 8.4. Machine Learning Complications in StentNet

The persistent challenge of inadequate sensory feedback impedes the progress of minimally invasive surgical procedures. While endoscopy has made some strides in addressing this issue, endovascular interventions like angioplasty or stenting still confront substantial obstacles. Traditional sensors relying on unobstructed lines of sight are ineffective in blood environments, rendering them unable to provide essential feedback. During stent deployment procedures, real-time feedback regarding the deployed stent’s condition is pivotal, as even a partially open stent can disrupt blood flow. Regrettably, no robust and noninvasive clinical solutions are currently available for monitoring stent deployment in real time.

In recent years, radio frequency (RF)-based sensors have garnered increasing attention as a potential solution. These sensors can detect the shape and composition of objects concealed from direct lines of sight. Notably, combining a 3D RF-based imaging sensor and a novel convolutional neural network (CNN) known as StentNet has emerged as a promising approach for monitoring stent deployment without the need for unobstructed lines of sight.

Conventionally, convolutional neural networks (CNNs) have been predominantly employed in classification tasks. For instance, the application of autoencoding CNNs has been instrumental in classifying fluorescent images of individual cells [208]. An important milestone in this domain is the groundbreaking research conducted by Sajjad et al. [209], wherein they introduced an innovative CNN-based multiclass classification model designed for precisely categorizing brain tumors. Moreover, CNNs have demonstrated substantial potential in medical image processing. In an enlightening study, Tang et al. [210] introduced a sophisticated deep-learning model that harnesses the power of CT images to automate the precise delineation of critical structures within the head and neck.

In parallel, the utilization of RF-based sensors has garnered significant attention, primarily due to their remarkable capability to penetrate obstacles. A recent publication [211] marked a milestone by introducing a pioneering medical imaging technique grounded in microwaves for detecting breast pathology. Additionally, RF-based data, when coupled with CNNs, were effectively employed for precisely segmenting ovarian structures in a separate study [212].

**Figure 18:**
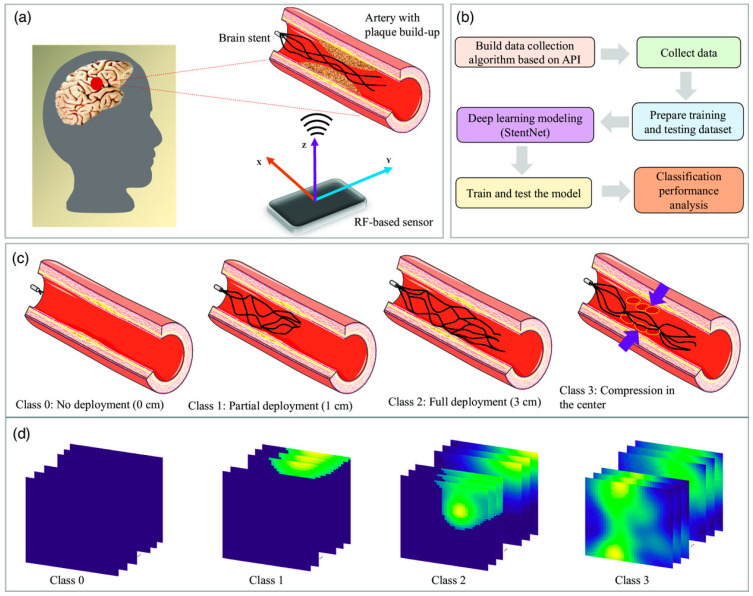
The stent deployment detection system, as elaborated in the research conducted by Xu et al. reproduced with permission [213]. Copyright 2020, John Wiley & Sons, Inc. (**a**) The experimental setup includes a stent and an RF-based sensor. The sensor is responsible for transmitting and receiving an amplitude-modulated signal, with the received signal being influenced by the shape of the stent. (**b**) The study workflow begins with data collection using the RF-based sensor for four distinct classes. A novel deep learning model, named StentNet, is introduced to detect stent deployment. (**c**) Data are collected in four different cases: no deployment (0 cm), partial deployment (1 cm), full deployment (3 cm), and full deployment with compression in the center. (**d**) Visualization of the four data classes shows the reflection power intensity, where darker colors represent higher reflection power.

In a comprehensive analysis of the stent deployment detection system, as expounded by Xu et al. [213], the authors underscore the strategic utilization of alterations in signal reflectance before and after stent deployment at the designated site. This innovative approach facilitates the evaluation of stent expansion and the detection of potential compression within the central region of the stent. Central to this methodology is deploying an RF-based sensor, the operational principles of which are vividly illustrated in Figure 18a,b as the experimental setup and workflow. The RF-based sensor adeptly performs its function by transmitting and receiving amplitude-modulated signals through linearly polarized broadband antennas. The workflow of this study adheres to a meticulously structured approach. Initially, data are systematically collected through the RF-based sensor, encompassing all four pertinent classes. Subsequently, an advanced deep learning model, denoted as StentNet, is introduced to detect stent deployment. The resultant representation of the radiated space post-stent deployment, characterized by variations contingent upon the stent’s specific configuration, is subsequently harnessed for precise classification purposes.

The data output derived from the RF-based sensor captures fluctuations in reflectance, and an algorithm, intricately integrated with an application program interface (API), is meticulously crafted to compile data across four distinct classes, which include a unique scenario: no deployment (0 cm), partial deployment (1 cm), full deployment (3 cm), and full deployment with compression at the center, as visually portrayed in Figure 18c,d. Darker hues correspond to higher reflection power, offering a visual means to comprehend the intrinsic characteristics of the dataset. This pioneering approach culminates in the introduction of a cutting-edge deep learning model named StentNet, painstakingly developed to facilitate the accurate and efficient detection of stent deployment.

## 9. Testing Methodologies for the Evaluation of Stents

### 9.1. In Vivo and In Vitro Testing Methodologies

The testing of stents relies on rigorous in vivo and in vitro methodologies to ensure safety, efficacy, and compatibility with the human body. These methods aim to mimic the conditions under which stents operate within blood vessels, evaluating their performance in restoring and maintaining vascular patency, as well as their long-term effects on the surrounding vascular tissue. In vivo stent testing involves expanding stents in an arterial vessel. Utilizing various animal models [214,215,216,217,218,219,220,221], in vivo studies offer insights into the biological response to stents, including healing and endothelialization, while addressing the ethical and translational challenges of animal-to-human applicability. Complementarily, in vitro testing, through advanced tissue-engineered models and blood-vessel mimics, allows for detailed examination of stent–endothelium interactions, thrombogenic potential, and drug efficacy in a controlled environment. This dual approach provides a comprehensive evaluation framework, integrating the physiological relevance of in vivo observations with the specificity and ethical ease of in vitro analyses; thus, it is crucial for refining stent design and ensuring successful clinical outcomes.

### 9.2. Common In Vitro Tests

In vitro testing offers a controlled, reproducible environment to assess the functionality and safety of stent designs before proceeding to in vivo studies; therefore, it plays a critical role in early-stage evaluations [222,223,224]. This section aims to review prevalent in vitro testing methodologies alongside recent advancements within this domain. By simulating the conditions of the human vascular system, in vitro tests provide valuable insights into the mechanical and biological performance of stents [225,226,227]. Essential mechanical evaluations include assessing fatigue resistance to confirm the stent’s capacity to withstand the heart’s repetitive motion and conducting corrosion tests on metallic stents to ensure their longevity and mitigate metal ion release [228]. For drug-eluting stents, additional drug-release kinetics need to be studied to establish optimal therapeutic profiles; thus, they comprise a fundamental part of in vitro characterization [229].

### 9.3. Hemodynamic Assessments

Biocompatibility stands at the forefront of in vitro testing to prevent adverse reactions. Preliminary tests typically include cytotoxicity assays [230,231], hemolysis tests [232,233], and protein adsorption studies [234,235] to ascertain that the stent material neither triggers hemolytic reactions nor hampers cell proliferation, which could increase post-implantation risks. Following mechanical and biocompatibility assessments, the focus typically shifts to evaluating hemodynamic effects. In vitro hemodynamic analysis employs artificial arteries to document changes in blood-flow dynamics induced by the stent, such as alterations in pressure, diameter, and flow rate [236]. Accurately simulating the physiological environment, including maintaining body temperature and appropriate pH levels with physiological saline or blood-like fluids, is crucial for precise hemodynamic impact analysis [237]. Advanced techniques such as particle image velocimetry [238] and computational models are instrumental in visualizing flow disturbances around stent struts [239] and predicting shear stress effects on endothelial cells [240]. These evaluations are vital for designing stents to minimize flow disruption and the associated thrombosis risk.

### 9.4. Testing for Thrombogenic Response

Several models have been reported to evaluate the thrombogenicity of stents. Combining integrated approaches, including in vivo, ex vivo, and computational analyses, Kolandaivelu et al. explored the influences of stent design, deployment, and drug-eluting coatings in thrombogenic response [241]. A modified Chandler loop system [242] was used to evaluate thrombosis associated with endovascular devices. This system utilized motor-controlled rotors accelerating blood-filled silicone loops to generate pulsatile flow simulating coronary-like hemodynamics. To model wall injury, loop segments were made reactive through incubation with bovine type I collagen solution, then rinsed with phosphate-buffered saline (PBS). Stents of different designs were deployed into these reactive segments under specific configurations (well-apposed, malposed, or overlapped), and thrombus formation was assessed. The testing results obtained using this model were compared to the in vivo porcine model where stents were deployed into coronary arteries using standard techniques. These values were then fed into a computation fluid dynamic simulation to model flow perturbations induced by stent struts of varying thickness within a simulated arterial lumen. The simulations were aimed at correlating computational predictions with the spatial distribution of clots observed in the experimental setups. This integrated approach allowed the authors to systematically investigate the factors influencing stent thrombogenicity from multiple perspectives, providing a comprehensive understanding of how stent design, deployment, and coatings interact with the vascular environment to affect thrombus formation.

**Figure 19:**
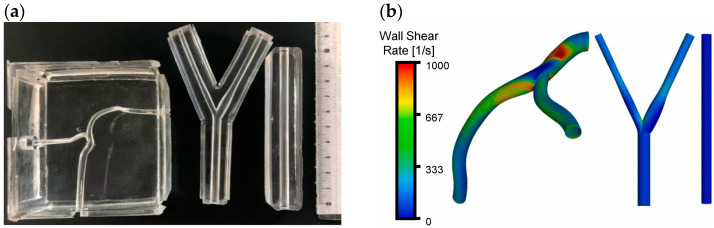
(**a**) macrofluidic flow chambers modeling the left anterior descending coronary (left), carotid (middle) and femoral (right) arteries, and (**b**) shear rate distributions within the models: extracted from CFD models. Reproduced with permission [243]. Copyright 2024, CellPress.

In a more recent study, Kern et al. introduce a novel macrofluidic model designed to study the thrombogenicity of stents used in clinical settings and to assist in developing less thrombogenic stents [243]. The model consists of a circular closed-loop system, as shown in Figure 19, featuring a silicone elastomer flow chamber that can mimic various human arteries (coronary, carotid, or femoral).

**Figure 20:**
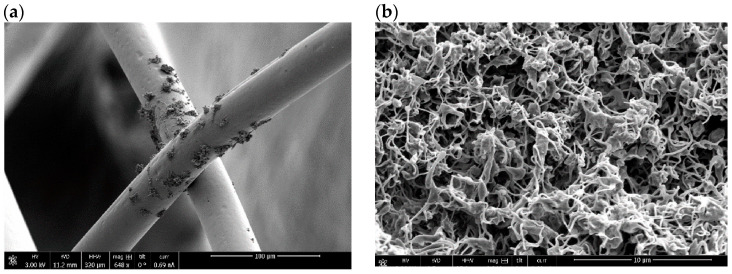
SEM images depicting platelet aggregates on a carotid stent after perfusion with blood for 1 h. (**a**) Aggregates are observed at the intersection of the stent meshes and (**b**) composed of tightly packed platelets. Reproduced with permission [243]. Copyright 2024, CellPress.

This system enables the insertion of actual stents and the study of their interaction with flowing blood, specifically focusing on platelet accumulation and thrombus formation shown in Figure 20. Blood perfused through the system is anticoagulated with hirudin to prevent coagulation and labeled with fluorescent markers for real-time observation of thrombus formation via video microscopy and further analysis with scanning electron microscopy. Through identification of thrombi formation composed of activated platelets, the study suggests that stent design, including strut thickness, number, and geometry, significantly influences thrombogenicity.

### 9.5. Endothelialization Evaluation

The long-term success of stent implantation not only depends on stenting techniques [158] and stent design but also hinges on its ability to promote endothelialization [244]. Stent endothelialization refers to the process by which endothelial cells, which line the interior surface of blood vessels, grow over and cover the surface of a stent implanted into a blood vessel [245]. This process plays a vital role in integrating the stent into the blood vessel and restoring the vessel’s natural lining. A complete and healthy endothelial layer reduces the risk of thrombus formation on the stent surface by providing a smooth and non-reactive barrier between the blood and the foreign material of the stent [246,247,248]. Endothelialization also facilitates the healing process of the vessel wall after the injury caused by stent implantation [249]. Furthermore, by covering the stent struts, endothelial cells can help prevent neointimal hyperplasia [250], restenosis caused by proliferation of smooth muscle cells, by maintaining a barrier to cell migration and proliferation.

Evaluating endothelialization involves seeding endothelial cells onto the stent surface and quantifying cell adhesion, proliferation, and the formation of a confluent endothelial layer, which is indicative of a healthy endothelium. Because of the stent’s complexity and the dynamic arterial compartments of the blood and vascular wall [251], there is no globally accepted in vitro testing method for analyzing endothelialization. A promising in vitro option for the evaluation of intravascular devices involves the use of blood-vessel mimics (BVMs). These are tissue-engineered constructs that simulate real blood vessels, offering a platform for preclinical testing to assess how endothelial cells react to devices such as coronary stents [252,253,254,255,256]. To create BVMs, tubular scaffolds made from materials such as expanded polytetrafluoroethylene (ePTFE), poly(L-lactide-co-caprolactone), silicone, and poly(lactic-co-glycolic) acid (PLGA) are combined with human vascular cells. The assembly is then cultured to develop a layer of cells inside the scaffold’s lumen. The cellular component primarily includes human endothelial cells and smooth muscle cells, which are obtained from either umbilical vein or coronary sources [252,253,256,257].

Initial iterations of BVMs typically featured straight channels constructed from silicone tubes, enhanced with extracellular matrix materials to support the adherence and proliferation of endothelial cells. For instance, Punchard et al. explored the effects of stent deployment on endothelial cells within a simulated coronary artery [253]. Their investigation employed an in vitro cardiovascular simulator designed to mimic the biomechanical conditions of coronary arteries. This approach utilized medical-grade silicone tubes as surrogate arteries, which were coated with fibronectin and inoculated with human umbilical vein endothelial cells (HUVECs). Following a 24-hour growth period, a bare-metal stent was inserted into these artificial arteries, which were then subjected to additional biomechanical conditioning to emulate physiological conditions. The study conducted gene expression analyses to evaluate the impact of stenting on HUVECs, specifically targeting markers linked to inflammation (E-Selectin, ICAM-1, and VCAM-1), apoptosis (Bax and Bcl-2), and cell proliferation (c-fos and Ki67). As expected, the expression of inflammatory genes increased significantly post-stent insertion, indicating a heightened inflammatory response. Meanwhile, an upregulation of pro-apoptotic gene expression alongside a reduction in anti-apoptotic gene expression was observed, suggesting an elevated apoptotic activity post-stent deployment, though direct evidence of apoptotic morphology was absent. Interestingly, proliferation markers such as c-fos transcription increased, suggesting a proliferative response, while Ki67 levels decreased, indicating reduced cell proliferation or a possible arrest in the cell cycle. The study demonstrated the potential of using tissue-engineered blood-vessel mimics within a controlled hemodynamic environment to better understand the cellular and molecular dynamics post-stent deployment.

In recent developments, BVMs have advanced from simplistic straight channels to employ more physiologically relevant, complex geometric designs. For example, Chavez et. al. [258] created a model to incorporate complex vessel geometries, such as bends (>45˚) and bifurcations. This BVM design utilized a polypropylene container for its primary chamber coupled with silicone tubing to facilitate fluid flow. HUVECs were cultured onto ePTFE scaffolds shaped into complex geometries. These scaffolds aimed to model the tortuous paths found in coronary arteries, with bends and bifurcations that challenge stent placement and endothelialization. A coronary stent was then deployed into a U-shaped BVM that had been pre-cultivated to establish an endothelial cell lining. After stent deployment, the BVMs were further cultivated, and the endothelialization process was monitored. Techniques such as histological analysis and scanning electron microscopy were employed to observe cell deposition and endothelialization, with a specific focus on identifying the reformation of an endothelial cell layer over the stent, a marker of successful re-endothelialization. This approach exemplifies a refined methodology for the investigation of stent endothelialization within an in vitro model, closely mimicking the physiological and anatomical conditions of human coronary arteries. Such advancements hold the promise of significantly enhancing the preclinical assessment of stents, thereby aiding in the refinement of stent designs to minimize the risk of restenosis and promote endothelial recovery.

Incorporating complex, biomimetic structures and pioneering biomaterials, recent efforts have led to the creation of vascular analogs for the preclinical evaluation of stents. Polydimethylsiloxane (PDMS), an elastomeric polymer, has been widely used in various biomedical applications due to its chemical stability, gas permeability, robust mechanical attributes, biocompatibility, remarkable optical clarity, and straightforward moldability [259,260]. In an innovative study, Weber et al. established a vascular mimic utilizing PDMS alongside endothelial cells derived from human induced pluripotent stem cells (hiPSCs) [261], thus achieving a model that more accurately reflects the human vascular system for stent assessment. The research aimed at enhancing stent–endothelium compatibility to address in-stent restenosis and the delay in post-implantation healing. The study demonstrated a functional endothelial layer within the PDMS model and allowed for the examination of inflammatory responses and the subsequent adhesion of immune cells to the endothelium under simulated physiological conditions. When testing stent endothelialization, the model showed a significant difference in the behavior of endothelial cells toward vitronectin-coated versus uncoated stents, highlighting the potential of surface coatings to enhance endothelial compatibility. This research presents a significant advancement in preclinical testing methodologies for vascular implants. The utilization of hiPSC-derived ECs in a biomimetic PDMS model represents a step toward personalized and more clinically relevant in vitro testing platforms. Such models can potentially reduce the reliance on animal testing by providing a human-specific system to evaluate the biocompatibility and hemocompatibility of new stent designs and materials.

The design and placement of stents can significantly affect the hemodynamic and mechanical environment of the artery after the procedure [156,262]. Modern in vitro and ex vivo models offer a sophisticated and biomimetic platform for assessing stent–host interactions. By closely mimicking human vascular conditions and allowing for the direct observation of stent–vessel interactions, these models represent a significant advancement in preclinical stent testing and design optimization. These models serve as valuable tools for both research and development, thus have the potential to enhance the understanding of host response and contribute to the innovation of safer, more effective stent design. Despite the robust framework of in vitro testing, these methods have limitations in fully replicating the complex nature of human biology. The ongoing evolution of these tests seeks to close the gap between in vitro findings and clinical outcomes, incorporating more sophisticated biological models and computational techniques to enhance predictability.

## 10. Challenges and Approaches

In this part, challenges in manufacturing and applications of stents and complementary approaches are discussed. We include more information about potential risks or complications associated with self-expanding metallic stents and strategies for mitigating these risks. Special attention is paid to AM of these devices by highlighting the progress and remaining issues. Common challenges in stenting include stent failure, tissue growth within the stent, recurrent vein occlusion, thrombosis, and vascular damage, which can be addressed by using a proper delivery mechanism, increasing radial stiffness and fatigue life, and reducing the strain of stent [3,78].

### 10.1. Manufacturing Challenges

**Figure 21:**
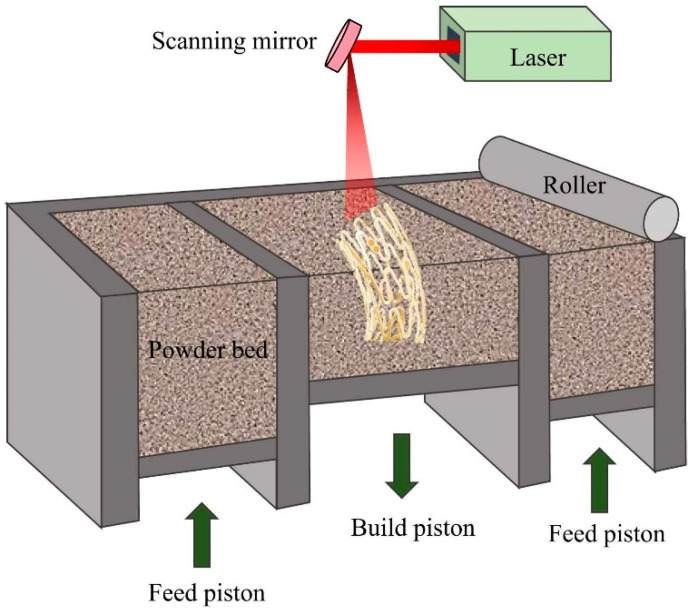
Schematic of laser powder bed fusion process to fabricate stent. Reproduced with permission [30]. Copyright 2022, Elsevier.

Nitinol stents are made by conventional methods, including photochemical etching, knitting, and coiling [30]. The different steps involved in these methods, such as machining, laser cutting, and shape setting, can result in material loss and increased cost. Moreover, in braided and knitted wire-based stents, filaments may intersect, increasing thickness [46]. Additive manufacturing, with the benefit of freedom of design in producing complex detailed geometries with high accuracy, as well as reducing time and cost, gained interest in medical applications, especially patient-specific stents [101]. Several additive manufacturing methods are categorized based on the feedstock form and energy source. Laser powder-bed fusion is the most commonly used technique for fabricating metallic implants and load-bearing devices [263]. As shown in Figure 21, this process uses powder as feedstock and a laser as the energy source to melt the powder and form the final part in a layer-by-layer process. However, there are some challenges in the fabrication of NiTi stents that are related to either the nature of AM itself or the material characteristics, such as compositional sensitivity, which can significantly affect the superelasticity and performance of the stent. Some SMAs (e.g., nitinol) can deform and repeatedly restore their original shapes and are ideal for peripheral stents; however, the difficulty in manufacturing these implants has dramatically limited their usage. For instance, Ni evaporation during the LPBF process alters the Ni/Ti ratio, leading to changes in transformation temperatures and producing too-rigid stents at body temperatures. Therefore, the process parameters must be carefully optimized for manufacturing of stents [107].

### 10.2. Stent Failure

In this part, failure mechanisms in stents and methods of addressing them will also be discussed. Currently, some PAD stents, including superficial femoral artery stents, have recently failed because of the reasons as follows: (1) complex motions of the artery leading to excessive mechanical loads on the stents and their failure to accommodate the desired shape change resulting in an artery occlusion; (2) disruption of the blood flow by stenting; and (3) mechanical failure during compression, bending, and torsion leading to artery damage and restenosis [54,77,264,265]. Stent placement in SFA is challenging due to its complicated biomechanical loading state and the varying loading in the different movements for example in the knee, and the stent should be able to adapt to this change in compression–tension forces and the resulting change in size of the artery [77]. Restenosis refers to the formation and growth of a tissue called neointima within 6 to 9 months of implementation that reduces the lumen size of an artery. Restenosis is caused by damage to the vessel wall and can cause chronic inflammation, leading to neoatherogenesis [54]. This complication is more pronounced in drug-eluting stents. Self-expanding stents have been shown to widen the stenosis for porcine coronary arteries [266].

### 10.3. Microbial Biofilm Formation

The medical stent is placed in the human body in contact with organs, tissues, and blood vessels. This can bring about some challenges. One of the main challenges of metal stents is the formation of microbial biofilm on the inner surface of the stent. Microbial biofilms are enclosed matrix of microorganisms that adhere to a surface and self-produce, which can lead to infection [267]. Moreover, bacteria lead to calcium and magnesium crystals forming on the outer surface of the stent, causing blockage and becoming a favorable site for microorganisms to adhere and develop. Formation of these biofilms on the surface of medical implants is one of the important concerns and it can even be lethal. In terms of manufacturing, there are some techniques to prevent the adhere and growth of microbic film on the surface of the stent. Surface treatments to increase roughness or make it hydrophobic are some examples of helpful approaches [48]. Toyokawa et al. [268] coated self-expandable metal stents with poly(2-methoxyethyl acrylate) and poly(3-methoxypropylacrylate) and observed that these coatings prevent the formation of conditioning film in the early stage of bacteria adhesion compared to conventional coating materials such as silicone and polyurethane. In another study, Lee et al. [269] improved a polymer coat by adding silver particles and reported enhanced behavior compared to silicon coating. In vitro studies show that this coating successfully prohibited biofilm formation on metal stents without releasing Ag from the coating. In vivo analyses indicate that the coating reduced the thickness of the biofilm. Another approach is to detect the microbial biofilm at its early stage of formation. Akhmetzhan et al. [270] used a bioelectrochemical technique on cobalt chromium drug-eluting stent. To validate this method, they performed biochemical characterization, and the results were consistent. This in vitro method can be used to evaluate the quality of the stent and to detect the formation of biofilms.

## 11. Outlook and Perspectives

### 11.1. Current Statistics and Outlook

The stents market is large and fast-growing due to increasing coronary artery diseases nowadays. Statistics from the Journal of Nursing and Health Studies show that one-fourth of men and one-sixth of women die from heart attacks in the United Kingdom. The American Heart Association also stated that 650,000 Americans die annually from coronary artery disease. Over 3 million stents are annually implanted worldwide, with the US and the UK having shares of around 600,000 and 50,000 stents, respectively. Each bare-metal and drug-eluting stent costs about EUR 500 and 2000, respectively. Due to yearly rising demands for stents, stents with faster production rates, higher quality, and lower cost will be competitive [271]. The global stent market was valued at USD 8.6 billion in 2021, with a vascular stent share of more than 92.1% and metal stents accounting for 6937.3 million. It expects a compound annual growth rate (CAGR) of 4% from 2022 to 2028 [272]. This growing market is due to the advancement in stent manufacturing and the production of high-accuracy stents. On the other hand, strict regulations and safety measurements hinder the growth of the stent market. The World Health Organization reported that cardiovascular diseases were among the significant causes of death, accounting for 32% of total global fatalities in 2019. According to research published by the National Library of Medicine in June 2021, coronary artery disease is responsible for 610 K fatalities a year, and it is the major cause of mortality in the US [273].

### 11.2. Market Data for Stents

In the stents market, Abbott Laboratories, Boston Scientifics, and Medtronic have a significant presence [273]. Several companies and manufacturers are producing stents. Table 5 provides a list of metal-stent manufacturers, along with the materials and techniques used.

## 12. Extended Summary

This paper provides readers with a unique outlook on the manufacturing, materials, characterization, and applications of stents. All the State-of-Art challenges, as well as future perspectives, are also addressed. Our review paper comprehensively considers the approaches mentioned earlier to give the reader a more detailed look at the manufacturing and processing of stents. This review paper covers some of the remaining gaps in the consideration of stents: (I) describing the challenges and the available approach to address them; (II) showing how to take advantage of the previously developed experiments, make them even, and design new stents with tailored properties; and (III) exploring future directions in the design and application of newly manufactured stents. In addition, multiple charts, tables, and SmartArt graphics are embedded into the entire manuscript to clarify the points mentioned in the text and to give the reader a better understanding and overview of the manufacturing/processing of stents. Although several papers have been recently published on reviewing the stents, this review paper meets the needs of the researchers working on stents by preparing them with a newly organized and broad outlook on manufacturing/processing of optimized stents. Future research should focus on further refining and expanding the manufacturing and processing techniques of stents to address current challenges. Researchers should build on the foundational experiments and insights highlighted in this review to design stents with tailored properties that meet specific clinical needs. Additionally, there is a need to explore new avenues in stent design, leveraging advanced technologies such as additive manufacturing, to create next-generation stents with enhanced performance and biocompatibility. By integrating comprehensive data analysis and multidisciplinary approaches, future studies can provide deeper insights to further develop stent technology.

## Figures and Tables

**Table 1:** Pros and cons of materials used for manufacturing metal stents [19,30,45].

Stent Material	Advantages	Disadvantages
316L SS	Widely used material due to its good mechanical properties and corrosion resistance	Non-MRI compatibility, poor visible fluoroscopic behavior, and allergic reactions in the body
NiTi (nitinol)	High corrosion resistance, Shape memory alloy (showing superelasticity, shape memory effect, and damping)	Ni release causing allergic reactions, nitinol stents do not present adequate radiopacity, crevice corrosion, and pitting
Co-Cr alloys	Radiopaque and MRI-compatible, high corrosion and wear resistance, superior radial strength and toughness, and suitable castability	Their plasticity and workability are inferior to those of stainless steel
Mg alloys	Biocompatible with good mechanical performance	The degradation products of Mg stents are toxic
Pt-Ir alloys	Excellent radiopacity, high corrosion resistance, and reduced thrombosis and neointimal proliferation	Lack of sufficient mechanical properties
Ta	Excellent radiopacity, MRI compatibility, and corrosion resistance in the human body	

**Table 2:** Comparison between the manufacturing methods of SEMSs [21,30,31,58,61,66,71].

		Methods		Materials	Advantages	Disadvantages
**Conventional Methods**	**Primary Manufacturing**	Casting		Mg alloys		Appropriate for simple shapes, restricted to materials with high ductility
		Conventional PM	CS	Stainless steel (SS316L), nitinol, Fe-Mn alloys		
			SPS	Nitinol, Fe-Ag, Fe-Au alloys		
			MIM	Fe	High production efficiency, good surface quality, high consistency	Difficult to processing
		Braiding		Stainless steel, Ta, Co-Cr alloys, Ni-Ti alloys	Low-cost, simple, and versatile continuous fabrication method with no material loss; producing stents with superior properties and no HAZ	High axial rigidity and length variations in the produced stents, poor radial stiffness, stent shortening problems in neighboring tough tissues, Limited to simple structure
		Knitting			Low-cost, simple, and versatile continuous fabrication method with no material loss; producing stents with superior properties and no HAZ	Low shortening ratio and compression resistance; biomechanical limitations; mismatch between their longitudinal flexibility and radial compliance with the artery
		Electroforming		SS316L, Fe, Fe-Mn, Fe-Zn	Low-cost, precise, and reproducible method, manufacturing products with complex shape and large size	Peeling of deposits from cathode, Limited materials so far
	**Secondary Manufacturing**	Photochemical etching		Stainless steel, nitinol, Co-Cr alloys	Low-cost, simple, rapid, and flexible method for stent manufacturing with no residual stress and burrs	Inappropriate for manufacturing 3D complex samples, non-uniform coating creation on the stent surface
		Micro-electro-discharge machining (μEDM)		Stainless steel, Mg alloys, Ti alloys	Producing stents with high surface quality and dimensional accuracy, and burr/dross-free	Limited to specific materials
		Laser cutting		SS316L, Co-Cr, Fe-Mn alloys	Low cost, High fabrication speed/precision/quality	HAZ
		Micro-milling		Pure Mg, Mg alloy	High process efficiency and accuracy	Burrs
		Welding		Stainless steel, nitinol	Low cost, No HAZ	Formation of brittle phases
**3D printing**		Selective laser melting (SLM)		NiTi alloys, Co-Cr alloys, Zn	Low-cost and fast fabrication, capable of producing stents with complex structures, improved geometrical accuracy, superior mechanical properties, high density and roughness, broad materials selection	Low strength of products, poor accuracy
		Electroforming		SS316L, Fe, Fe-Mn, Fe-Zn	Low-cost and precise manufacturing method	Limited materials so far

**Table 3:** List of SEMSs manufactured by different methods [30].

Methods	Name	Manufacturer	Material	Stent Form	Geometry
Photochemical etching	Endotex		Nitinol	Sheet	------
	aSpire	Vascular Architects	Nitinol	Sheet	Coil
Braiding	Wallstent	BSC	Co-Cr alloy	Wire	Braided
Knitting	ZA	Cook	Nitinol	Wire	Knitted
Coiling	Symphony	BSC	Nitinol	Wire	Welded coil
	Esophacoil	InStent	Nitinol	Ribbon	Coil
	IntraCoil	IntraTherapeutics	Nitinol	Wire	Coil

**Table 4:** Summary of the designs of stents with their strengths and limitations.

Design		Material	Stent Form	Application	Advantages	Disadvantage
Coiled		Metallic wire	Balloon-expandable	Successful in nonvascular: prostate and urethral	High flexibility	Limited radial strength, low expansion capability, significant elastic recoil, and a heightened risk of restenosis
				Not Successful in vascular	Large size	
Slotted tube		Metal tubes, followed by laser cut	Main available stent in the market		Impressive radial strength	Limited flexibility and deliverability
Tubular mesh (woven)		Wires	One or more wire strands, self-expanding and balloon-expandable (mainly SEMS)	Urological, gastrointestinal, and airway applications	Extensive coverage and minimal expansion, robust mechanical support to the arteries	
	Fiber-based	Fibers	Production by knitting and braiding		Easy to modify to enhance their biocompatibility, exceptional mechanical properties	
	Braided	Fiber, wire				Limited flexibility and a tendency for the edges to fray
	Knitted	Wrap knit			Natural flexibility due to their interconnected looped design, easily removable in the form of a wire	
		Weft knit				
Covered		Fully or partially covered SEMS		For esophageal strictures	Prevent excessive tissue growth around the wire meshes	Risk of granulation tissue forming at the exposed ends of the stent and tissue ingrowth through the disrupted covering
Closed-cell					Greater radial strength, more resistant to the growth of tumors or excessive tissue growth inward, longer patency	Less flexible
Open-cell		Periodic connections from peak to peak, from peak to valley, and from mid-step to mid-step			Longitudinal flexibility, more pliable, reduced surface area, neointimal reaction, and arterial injury, improved access to side branches, enhanced conformability, shortening ratio of zero	Lower radial strength, higher plaque prolapse
Helical pattern					Flexibility, few or no internal connection points	Lacking longitudinal support, possible irregular cell sizes after deployment

**Table 5:** List of stent manufacturers.

Company Name	Material	Manufacturing Process	Cost [274]
Abbott Laboratories (USA)	CoCr [275]	-	USD 100
amg International GmbH (Germany)	CoCr, NiTi [276]	-	-
Bard Angiomed [46]	NiTi	Laser cut tube	-
Boston Scientific (USA) [277]	PtCr stainless steel [275]	-	USD 75–1400
Biotronik (Germany)	NiTi	-	USD 450
Cook Medical (USA) [46]	NiTi	Knitted wire	USD 100
CSIRO (Australia)	NiTi [278]	3D printing	-
Medtronic (USA)	CoCr [275]	-	USD 150–1700
Medicorp Inc. (USA) [46]	NiTi	Braided wire	-
Nano Therapeutics Pvt. Ltd. (India)	CoCr [279]	-	-
Norman Noble, Inc. (USA)	NiTi [280]	Laser cutting	-
Optimed Medizinische Instrumente GmbH (Germany) [281]	NiTi	Braided	-

## Data Availability

No data were used for the research described in the article.
